# Study on the intracellular adaptative mechanism of *Acidithiobacillus caldus* MTH-04 to NaCl stress

**DOI:** 10.1186/s12934-023-02232-w

**Published:** 2023-10-25

**Authors:** Min Li, Jianping Wen

**Affiliations:** 1 Key Laboratory of Systems Bioengineering, School of Chemical Engineering and Technology, Tianjin University, Tianjin, China; 2 Collaborative Innovation Center of Chemical Science and Engineering, Tianjin University, Tianjin, China; 3 Frontier Science Center of Ministry of Education, Tianjin University, Tianjin, China

## Abstract

To understand the adaptive mechanism of bioleaching microorganism *Acidithiobacillus caldus* MTH-04, its physiology and metabolic changes at the transcriptional level were systemically studied. The results of growth curves, SO_4_^2−^ content, pH and flow cytometry analyses indicated that the higher the NaCl concentration, the more the strain was inhibited. The transcriptome response of *A. caldus* to elevated NaCl concentrations included changes in carbon flux, elevated glutathione synthesis, alterations in cell wall and membrane composition, the down-regulation in genes involved in flagellar synthesis and rotation, the reduced energy generation through sulfur oxidation, and the up-regulation in genes involved in DNA and protein repair. Based on the transcriptome results, the effects of proline and glutathione on NaCl adaptation in *A. caldus* were analyzed separately. We found that either the exogenous addition of proline and glutathione or the intracellular overexpression of the enzymes responsible for the synthesis of these two substances contributed to the enhancement of the adaptive capacity of *A. caldus* under NaCl stress. The findings offer insight into the design of chloride-based techniques for the bioprocessing of minerals.

**Supplementary Information:**

The online version contains supplementary material available at 10.1186/s12934-023-02232-w.

## Introduction

Halotolerant/halophilic acidophiles are of interest due to their potential in biomining in situations where the supply of freshwater is limited and seawater could be used instead [1]. However, the presence of ions, including sodium and sulfate ions, in seawater can lead to osmotic stress. Chloride ions represent a major challenge as they can penetrate cell membranes, acidifying the cytoplasm, leading to reduced cell growth and, ultimately death [2]. However, while sodium chloride has detrimental effects on acidophiles, it is possible that chloride might improve leaching efficiency. For example, *Sulfobacillus thermosulfidooxidans* strain Cutipay shows increased bioleaching of chalcopyrites when chloride ions are present [3]. Chloride is also known to improve the porosity and crystallinity of sulfur derived from chalcopyrite leaching, improving the access of the oxidant to the surface of the mineral [4].

In addition, it has been shown that the chemical Cl^−^ leaching drives chalcopyrite dissolution in water with high levels of salinity [5]. This chemical reaction needs to be supported by the introduction of designed microbial activity. The introduction of halophilic Fe-oxidizers is able to increase the redox potential (E_h_), thereby discouraging the Cl^−^ leaching reaction. The exploitation of halophilic S-oxidizers could assist in the maintenance of optimal low E_h_ ranges, together with providing acidic reactions for sustaining the proton-consuming Cl^−^-leaching reaction of chalcopyrite. Thus, the use of S-oxidizers should be considered when using water with high salinity, such as seawater.

*Acidithiobacillus caldus* (*A. caldus*) is a *r-Proteobacterium* with moderate thermoacidophilic and obligate chemolithotrophic properties. It grows optimally between temperatures of 40 and 45 °C and pH conditions between 2 and 2.5, and has been found to be the dominant sulfur-oxidizing microorganism for biomining [6]. In this process, it oxidizes both elemental sulfur and reduced inorganic sulfur compounds (RISCs) to increase the acidity necessary for biomining and removes additional elemental sulfur that could reduce ore oxidation [7]. *A. caldus* has been found to grown in the presence of high NaCl, and adaptations of its cell membrane were observed [1]. Halotolerant and halophilic bacteria show various adaptations to high-salt conditions, including (i) high potassium levels in the cytoplasm, cytoplasmic osmo-protectants for the maintenance of turgor pressure, (ii) membrane adaptations, and (iii) increased concentrations of acidic amino acids on protein surfaces, promoting the solubility of the protein [8]. Although there have been some investigations into adaptations to high salt in acidophiles [9, 10], a more comprehensive understanding of the occurrence, diversity and conservation of these mechanisms in acidophilic prokaryotes and their relevance in halotolerance is currently lacking.

The principal objective of the present investigation was to elucidate molecular mechanisms underlying the adaptation of *A. caldus* to hyperosmotic conditions. Therefore, we used comparative transcriptomics, exogenous additions, genetic modifications and other methods to elucidate the adaptive mechanisms of *A. caldus* under NaCl stress.

## Materials and methods

### Bacteria, plasmids, and culture conditions

Table 1 provides details of the bacterial strains and plasmids. The strain *Acidithiobacillus caldus* (*A. caldus*) MTH-04 was grown at 40 °C in Starkey medium containing CaCl_2_·2H_2_O (0.25 g/L), (NH_4_)_2_SO_4_ (2 g/L), MgSO_4_·7H_2_O (0.5 g/L), KH_2_PO_4_ (3 g/L), FeSO_4_·7H_2_O (0.01 g/L). H_2_SO_4_ was used for adjustment of the pH to 2.5. After autoclaving, 8 g/L of powdered sulfur (three consecutive steam sterilizations at 105 °C for 3 h) was added. *A. caldus* was added to 150 ml of Starkey-S^0^ medium and shaken at 150 rpm.

**Table 1:** Bacterial strains and plasmids used in this study

Strains/Plasmids	Dscription	Source or references
*A. caldus* MTH-04	Isolated from Tengchong, Yunnan Province, China; NCBI accession number: NZ_CP026328.2	China Center of Industrial Culture Collection [11]
AC00	*A. caldus* MTH-04 harboring pJRD215-Ptac	This study
ACP1	*A. caldus* MTH-04 harboring pJRD215-Ptac-proA	This study
ACP2	*A. caldus* MTH-04 harboring pJRD215-Ptac-proB1	This study
ACP3	*A. caldus* MTH-04 harboring pJRD215-Ptac-proB2	This study
ACP4	*A. caldus* MTH-04 harboring pJRD215-Ptac-proC	This study
ACP5	*A. caldus* MTH-04 harboring pJRD215-Ptac-yggS-proC-yggT-DUF167	This study
ACG1	*A. caldus* MTH-04 harboring pJRD215-Ptac-gshA	This study
ACG2	*A. caldus* MTH-04 harboring pJRD215-Ptac-gshB	This study
ACG3	*A. caldus* MTH-04 harboring pJRD215-Ptac-gshAB	This study
Plasmids		
pJRD215	Kmr, Smr; IncQ, Mob+	Laboratory stock
pJRD215-Ptac	Smr, Kmr; IncQ, Mob+; Ptac	This study
pJRD215-Ptac-proA	Smr, Kmr; IncQ, Mob+; Ptac; proA	This study
pJRD215-Ptac-proB1	Smr, Kmr; IncQ, Mob+; Ptac; proB1	This study
pJRD215-Ptac-proB2	Smr, Kmr; IncQ, Mob+; Ptac; proB2	This study
pJRD215-Ptac-proC	Smr, Kmr; IncQ, Mob+; Ptac; proC	This study
pJRD215-Ptac-yggS-proC-yggT-DUF167	Smr, Kmr; IncQ, Mob+; Ptac; yggS-proC-yggT-DUF167	This study
pJRD215-Ptac-gshA	Smr, Kmr; IncQ, Mob+; Ptac; gshA	This study
pJRD215-Ptac-gshB	Smr, Kmr; IncQ, Mob+; Ptac; gshB	This study
pJRD215-Ptac-gshAB	Smr, Kmr; IncQ, Mob+; Ptac; gshA-gshB	This study

### Physiological experiment

Physiological properties of *A. caldus* were determined by measuring growth curves, SO_4_^2−^ content, pH, and cell morphology. The growth curve of *A. caldus* was obtained by measurement of the OD_600_ values of the culture broth using a microplate reader (SpectraMax 190, Molecular Devices). The pH of the culture broth was measured using a pH meter (PHSJ-4 F, INESA Scientific Instrument Co. Ltd, China). The final product of *A. caldus* using S^0^ is SO_4_^2−^, so the SO_4_^2−^ content is used to characterize the sulfur oxidation ability of the strain. Barium chromate spectrophotometry was used to determine the SO_4_^2−^ content of the culture broth, see the details in Additional file 1: Text S1. Cell morphology, such as cell granularity and cell size, was characterized by flow cytometry. Specifically, the forward scatter (FSC) detector and side scatter (SSC) detector of a flow cytometer (FACSArial III, BD, USA) equipped with a 70 μm nozzle were used to detect the size and granularity of cells, respectively. The more complex the granular structure in the cell, the larger its SSC. The larger the cell, the larger its FSC. 10^5^ cells were counted per sample and data were analyzed using the software FlowJo_V10 software.

### RNA-seq and data analysis

After culturing *A. caldus* to the late logarithmic phase in Starkey-S^0^ liquid medium containing low salt (0 mM NaCl) and high salt (600 mM NaCl), culture was centrifuged (6000 rpm, 10 min, 4 °C) and the bacteria were collected and quickly frozen with liquid nitrogen. RNA extraction and sequencing were conducted by Majorbio Co., Ltd. (Shanghai, China). Total RNA was extracted from six samples (three per group) with TRIzol (Takara Bio, Dalian, China), following the provided directions. The quality and concentrations were assessed using electrophoresis and absorbances using a NANOdrop ND-2000 spectrophotometer (Thermo Scientific, Waltham, MA, USA) with integrity examined with a microfluidic assay on the Bioanalyzer (Agilent Technologies, Inc., Santa Clara, CA, USA). The rRNA depletion from total RNA was performed using the RiboCop rRNA Depletion Kit for Mixed Bacterial Samples (Lexogen, USA) and then all mRNAs were cleaved into short fragments by adding fragmentation buffer. These short fragments were used as templates to synthesize cDNA double strands. When synthesizing the second strand of cDNA, dUTP was used instead of dTTP to make the bases contain A/U/C/G. The second-strand cDNA was then digested with UNG so that the library contained only the first-strand cDNA. The library construction process described above was performed according to the instructions of Illumina^®^ Stranded mRNA Prep, Ligation from Illumina (San Diego, CA). After amplification with Phusion DNA Polymerase (NEB) and quantification with TBS380, the paired-end RNA-seq library was sequenced using the Illumina Novaseq 6000 (Illumina Inc., San Diego, CA, USA).

After obtaining the Read Counts number of the genes, differentially expressed genes (DEGs) between the groups were identified using DESeq2, using the criteria of adjusted p value ≤ 0.05 and |Log_2_Fold Change| ≥ 1. The functions of the genes were examined using the Swiss-prot, NR, Pfam, COG and GO databases. Goatools was used for GO enrichment and KEGG PATHWAY enrichment was performed using R scripts.

### qRT-PCR verification

Fourteen DEGs (seven up-regulated and seven down-regulated) were selected for verification of the RNA-seq data and appropriate primers (Additional file 1: Table S1) for qRT-PCR analysis were designed. The *map* gene, encoding type I methionyl aminopeptidase, was used for normalization. Total RNA was reverse-transcribed to cDNA using the StarScript III All-in-one RT Mix with gDNA Remover Kit (GeneStar, China) and quantified by qPCR using the PerfectStart Green qPCR SuperMix Kit (TransGen, China). Gene expression levels were calculated by 2^−△△ct^.

### Genetic manipulations and confirmation of transformed strains

The Ptac-F and Ptac-R primers were annealed to form double-stranded DNA, which was then ligated to pJRD215 digested with SaII and KpnI to form pJRD215-Ptac. The pJRD215-Ptac plasmid was digested with XbaI and EcoRI, and the gene sequences for synthesizing proline and glutathione were separately inserted after the tac promoter. The genes for synthesizing proline are *proB1*/*proB2* encoding glutamate 5-kinase, *proA* encoding glutamate-5-serialdehydrogenase, and *proC* encoding pyrroline-5 carboxylate reductase. In addition, a yggS-proC-yggT-DUF167 operon sequence was placed behind the tac promoter. The genes for synthesizing glutathione are *gshA* encoding glutamate-cysteine ligase and *gshB* encoding glutathione synthetase. For example, the *proA* gene from *A. caldus* was amplified from genomic DNA with primer pair proA-F/proA-R and purified. This was followed by combination of the PCR fragment with the digested pJRD215-Ptac plasmid using T4 DNA ligase (ABclonal, China) to generate pJRD215-Ptac-proA. Other plasmids were constructed in the same way as pJRD215-Ptac-proA. The construction principle of these nine recombinant plasmids is shown in Additional file 1: Figs. S3 and S4.

The constructs were transfected into *E. coli* DH5α and the sequences were verified by Genewiz Biotechnology Ltd. (Suzhou, China). Additional file 1: Table S2 lists the primers used. These constructed plasmids were conjugated from *E. coli* SM10 to *A. caldus* MTH-04 according to a published protocol [12]. After this step, positive clones carrying the corresponding plasmids were verified by colony PCR with primer pair 215MCS-F/215MCS-R. The colony PCR results of these successfully constructed strains are shown in Additional file 1: Text S2 and Text S3. The strain containing the plasmid pJRD215-Ptac was designated AC00, which also served as the control strain. The strains containing plasmids pJRD215-Ptac-proA, pJRD215-Ptac-proB1, pJRD215-Ptac-proB2, and pJRD215-Ptac-proC, and pJRD215-Ptac-yggS-proC-yggT-DUF167 for overexpressing proline synthesis genes were designated ACP1 ACP2, ACP3, ACP4, and ACP5, respectively. The strains containing plasmids pJRD215-Ptac-gshA, pJRD215-Ptac-gshB, and pJRD215-Ptac-gshAB for overexpressing glutathione synthesis genes were designated ACG1 ACG2, and ACG3, respectively.

### Assessment of intracellular ROS and glutathione contents

For glutathione measurement, AC00, ACG1, ACG2 and ACG3 cells were collected and washed once with Starkey medium with 300 mM NaCl, followed by washing with PBS (pH 7.4), centrifugation and assayed using the Reduced Glutathione (GSH) Content Assay Kit (Solarbio, China).

For measurement of ROS contents, strains were collected and washed once with Starkey medium with 300 mM NaCl. After washing in PBS, cells were treated with 10 µM 2′,7′-dichlorodihydrofluorescein diacetate (H_2_DCFDA) indicator in DMSO for 30 min at 25 °C in the dark, followed by further washing in PBS to remove the unbound indicator. Fluorescence (excitation, 488 nm; emission,525 nm) values were measured using a multifunctional microplate reader (INFINITE E PLEX, TECAN, Austria) and normalized by cell density.

## Results and discussion

### Physiological properties of ***A. caldus ***at different NaCl concentrations

**Fig. 1:**
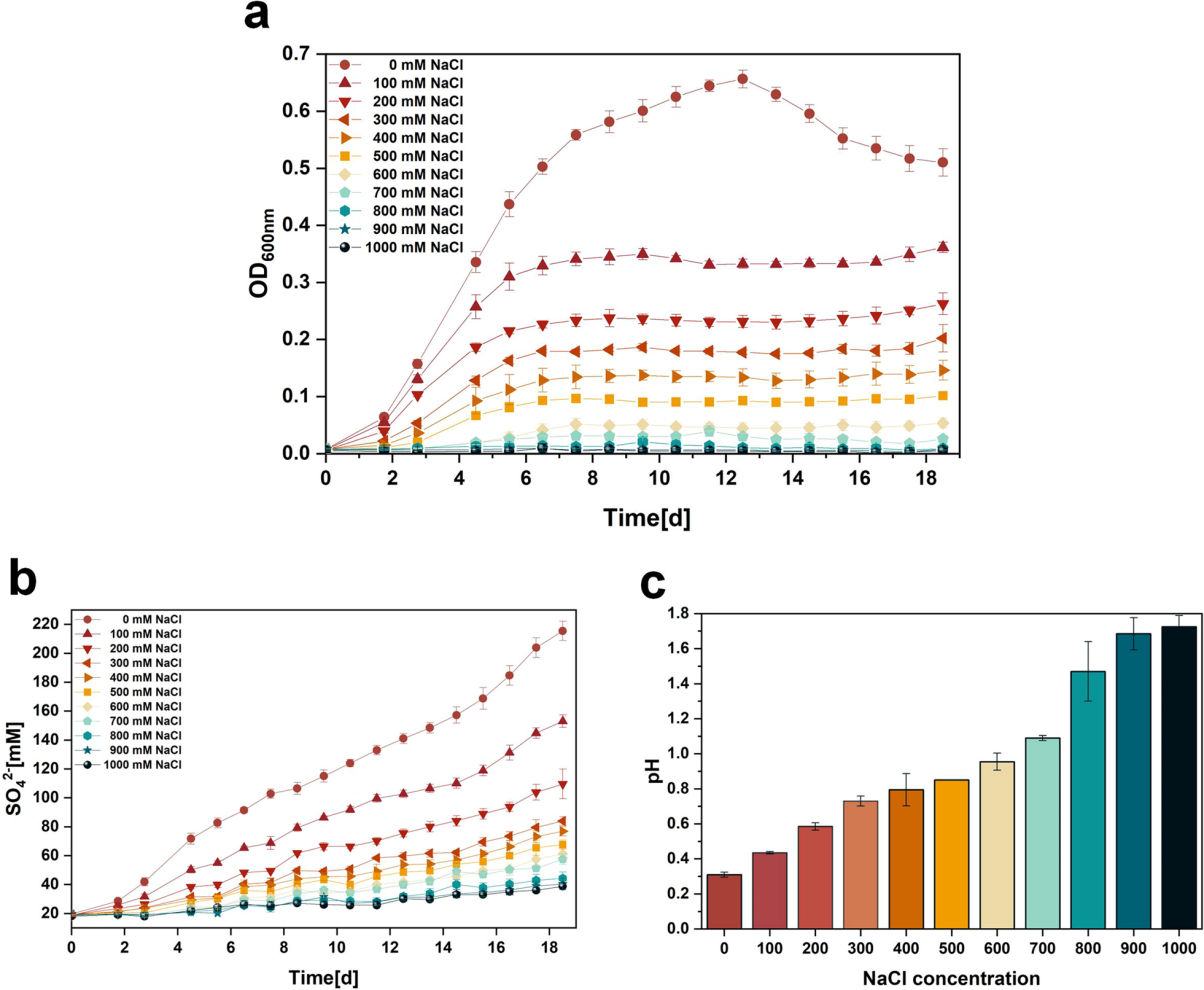
**a** OD_600_, **b** SO_4_^2−^ content and **c** final pH of culture broth of *A. caldus* at different NaCl concentrations

Strains were cultured in Starkey-S^0^ liquid medium with varying concentrations of NaCl (0–1000 mM) and the effect of NaCl on the strains was assessed by growth curves, SO_4_^2−^ content, pH and cell morphology. Figure 1a shows that the growth of the strains at different NaCl concentrations differed significantly from day 2 onwards. Specifically, higher salt concentrations inhibited the strain more. The effects of different NaCl concentrations on the strains included lower biomass and earlier stabilization period compared to 0 mM NaCl. When the NaCl concentration was higher than 800 mM, we did not observe significant growth of the strain. Therefore, the minimum inhibitory concentration of NaCl for *A. caldus* was 800 mM. Figure 1b shows that higher NaCl concentrations were associated with lower SO_4_^2−^ contents in the culture broth. The SO_4_^2−^ content of the culture broth usually corresponds to the pH, i.e. the higher the SO_4_^2−^ content, the lower the pH. Correspondingly, Fig. 1c shows that the higher the concentration of NaCl, the higher the final pH of the culture broth. Therefore, NaCl significantly inhibited the growth and sulfur oxidation capacity of *A. caldus*, which is highly unfavorable for bioleaching operations.

**Fig. 2:**
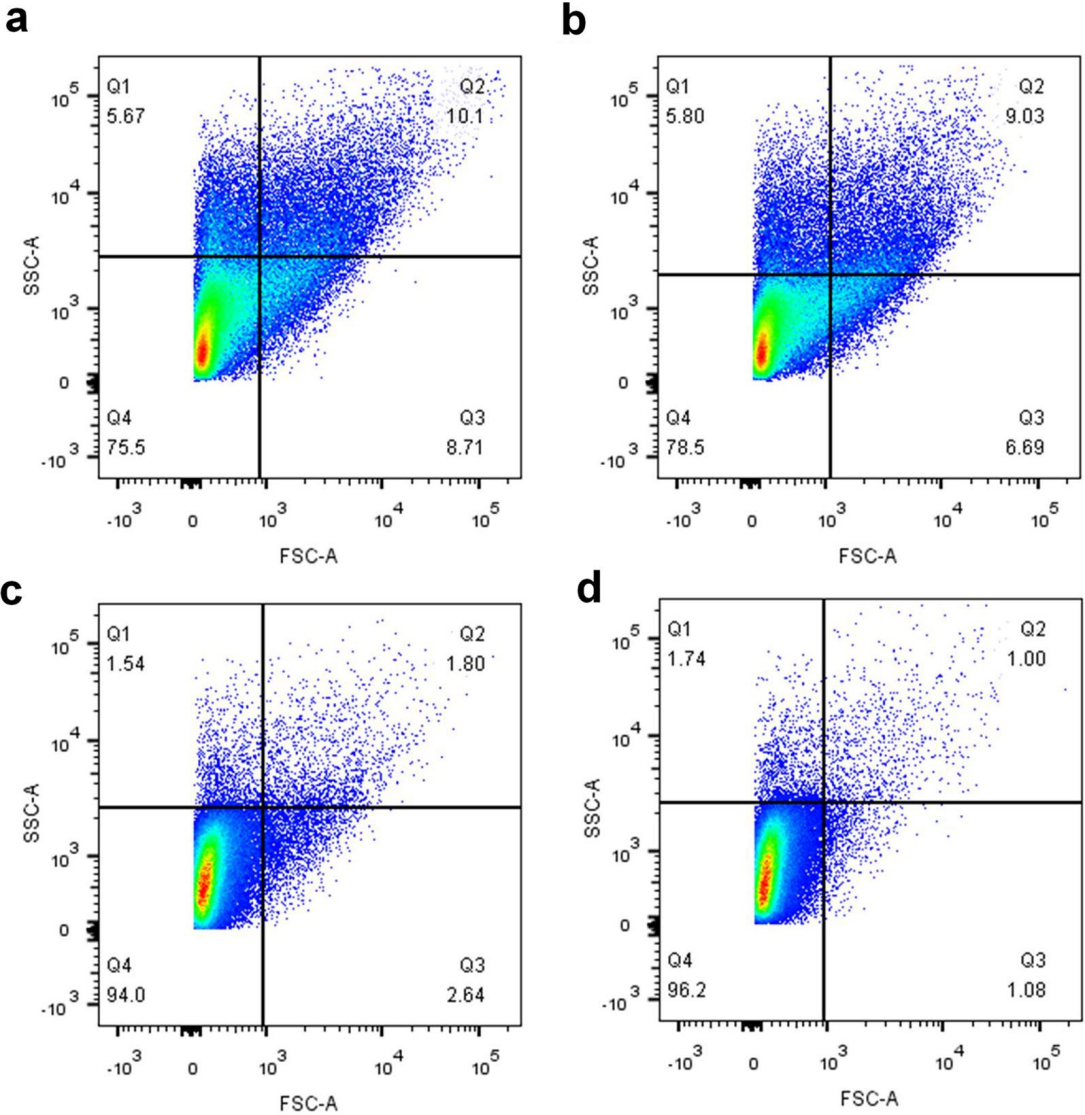
Cell size and granularity at different NaCl concentrations. **a** 0 mM, **b** 100 mM, **c** 300 mM, and **d** 500 mM NaCl.

In addition, we observed the cell morphology of *A. caldus* at 0, 100, 300, and 500 mM NaCl concentrations. Marked differences in both cell sizes and granularity were found. As shown in Fig. 2, most of the cells were distributed in the Q4 area, and their proportion was 75.5%, 78.5%, 94%, and 96.2% in sequence as the NaCl concentration gradually increased. Cells with smaller volumes and less intracellular particulate matter gradually increase, which may be due to cells losing more water because of elevated osmotic pressure.

### Transcriptome analysis under NaCl stress

#### Screening of DEGs

**Fig. 3:**
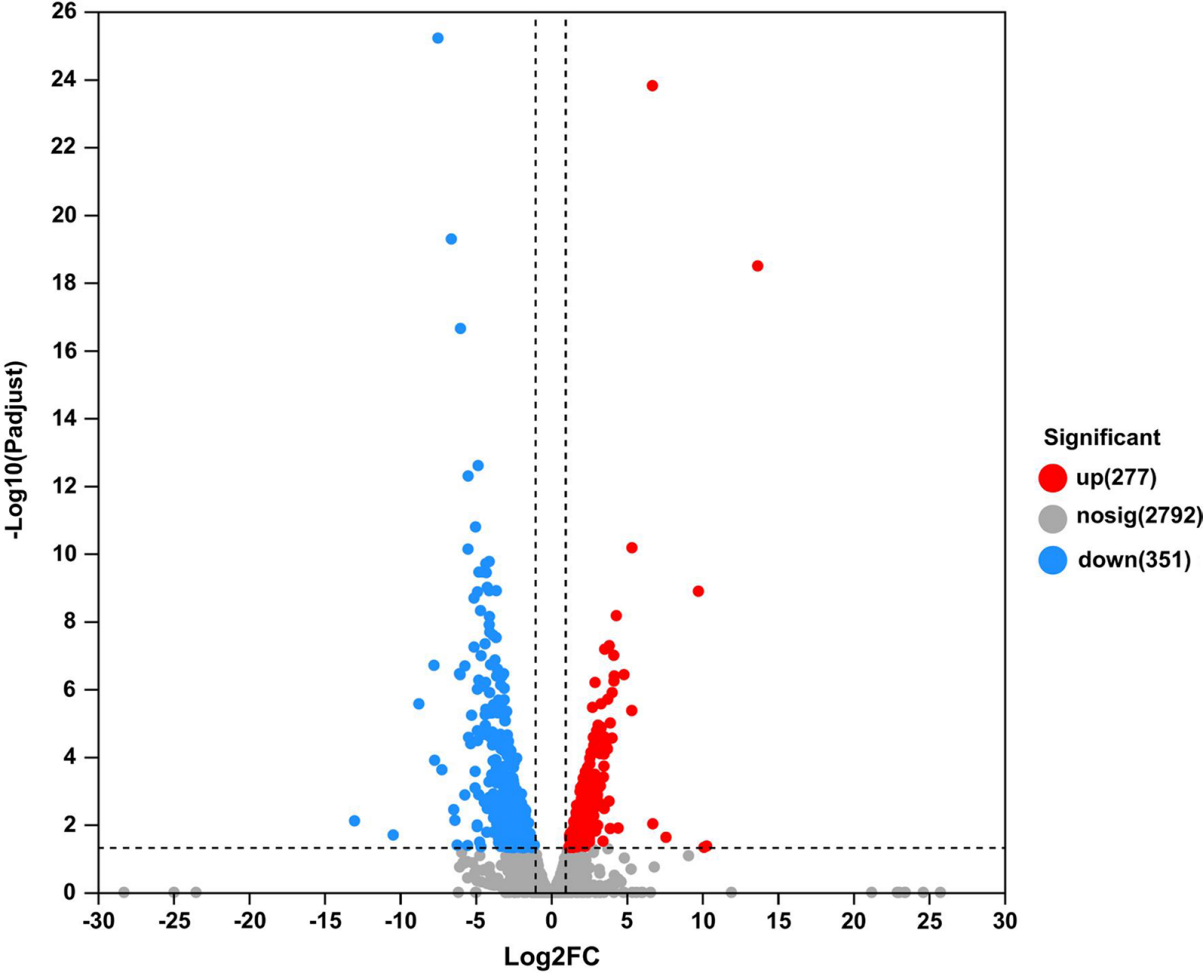
Volcano plots of DEGs under high salt (600 mM NaCl) and low salt (0 mM NaCl) conditions. The horizontal coordinate is the fold change value (FC value) and the vertical coordinate is the statistical test value (p value). Red dots represent up-regulation, while blue represents down-regulation, and gray indicates no significant change

**Fig. 4:**
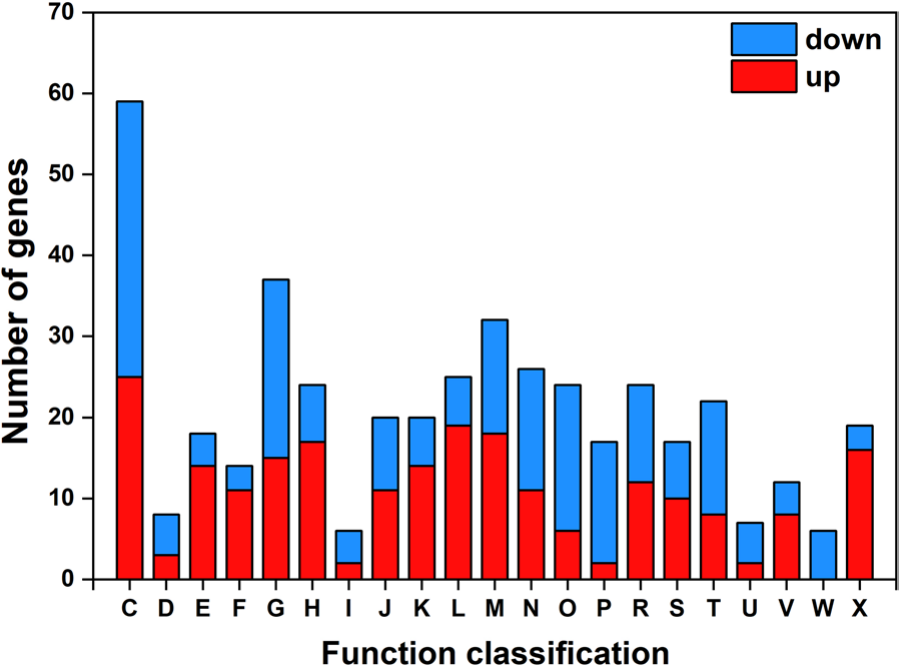
COG annotation analysis of DEGs

The transcriptomic data were analyzed to identify genes and mechanisms involved in the adaptation to NaCl stress. We analyzed the differences in gene expression under 600 mM NaCl and 0 mM NaCl conditions. As shown in Fig. 3, 628 DEGs were detected in group 600 mM NaCl vs. 0 mM NaCl. Of these, 277 were observed to be up-regulated while 351 were down-regulated. DEGs accounted for 18.36% of all detected genes, indicating that high salt stress greatly affected the strain. For subsequent analysis, the DEGs were classified by COG, as detailed in Additional file 1: Table S3. Figure 4 shows the COG annotation analysis of the DEGs. DEGs are classified in descending order of distribution number as energy production and conversion (C), carbohydrate transport and metabolism (G), cell wall/membrane/envelope biogenesis (M), posttranslational modification, protein turnover, chaperones (O) and mobilome (X). Specifically, the number of DEGs was higher in C, G and M with 59, 37 and 32 respectively.

#### KEGG, and GO enrichment analyses of DEGs

**Fig. 5:**
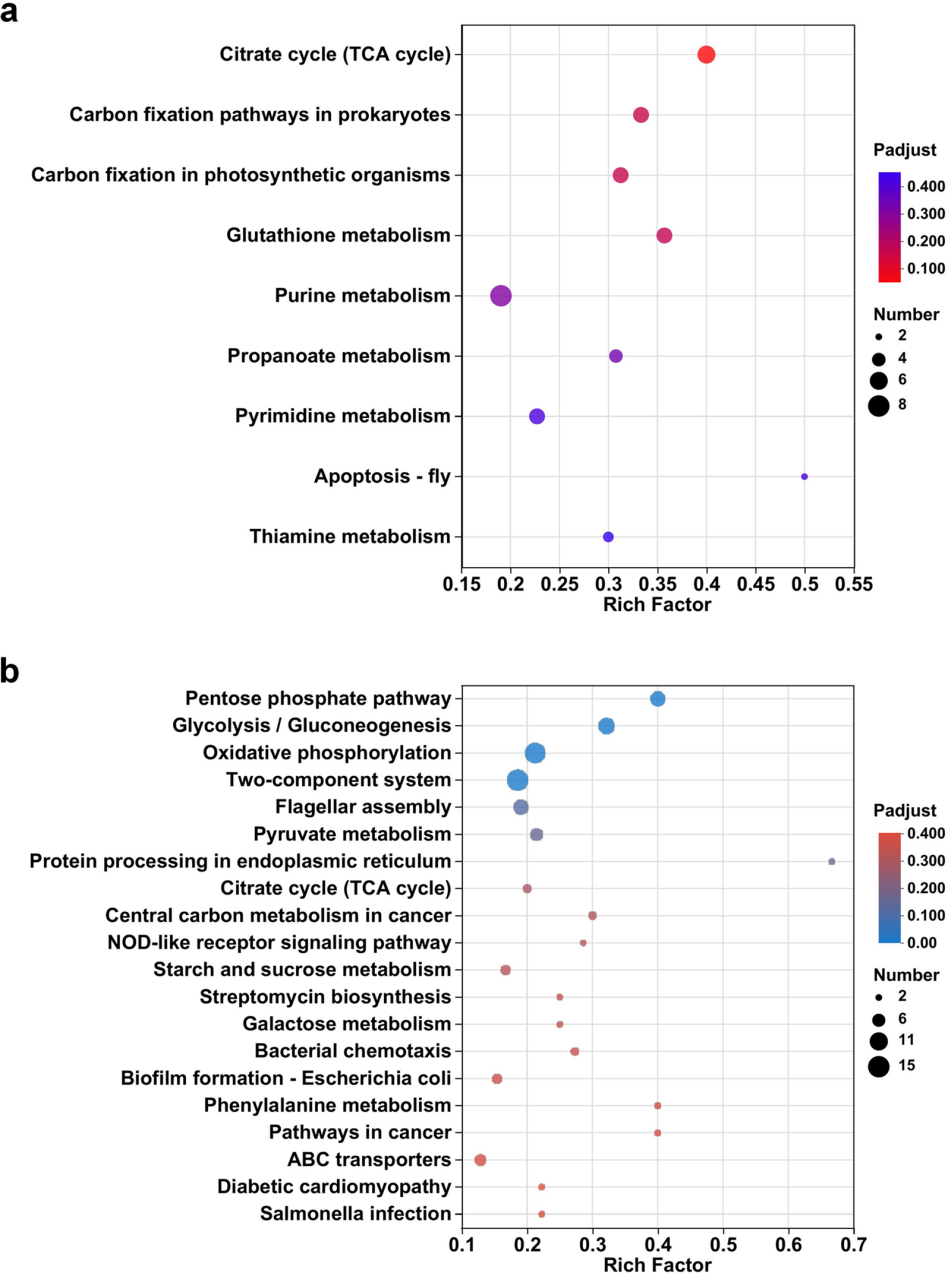
Bubble plots of KEGG enrichment analysis of **a** up-regulated and **b** down-regulated DEGs

**Fig. 6:**
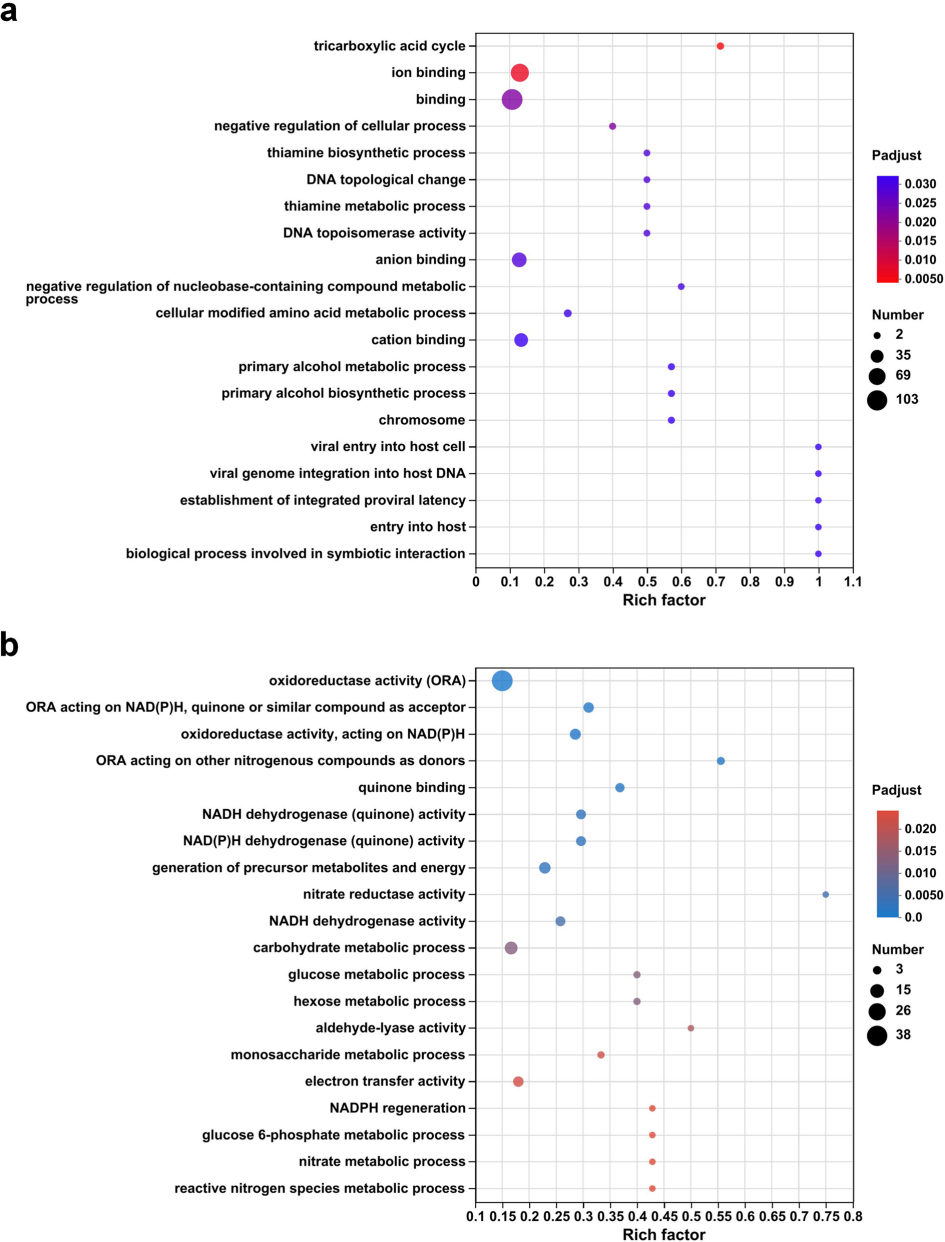
Bubble plots of GO enrichment analysis of **a** up-regulated and **b** down-regulated DEGs

KEGG and GO enrichment analysis can visually reflect the metabolic pathways involved in DEGs. KEGG pathway analysis (Fig. 5) showed that up-regulated genes were significantly enriched in the citrate cycle (0.4), glutathione metabolism (0.357) and carbon fixation pathways in prokaryotes (0.333), while the pathways with higher rich factor of down-regulated genes were pentose phosphate pathway (0.4) and glycolysis/ gluconeogenesis (0.321). These pathways with high rich factor are mainly associated with central carbon metabolic pathways. As shown in Fig. 6, GO enrichment analysis revealed that the pathways with higher significance of up-regulated genes were tricarboxylic acid cycle, ion binding and binding, while the pathways with higher significance of down-regulated genes were oxidoreductase activity, quinone binding, NADH dehydrogenase (quinone) activity and NAD(P)H dehydrogenase (quinone) activity. We found that many of the down-regulated genes were involved in terms related to energy metabolism, such as oxidoreductase activity, quinone activity and dehydrogenase activity.

#### Carbohydrate transport and metabolism

**Fig. 7:**
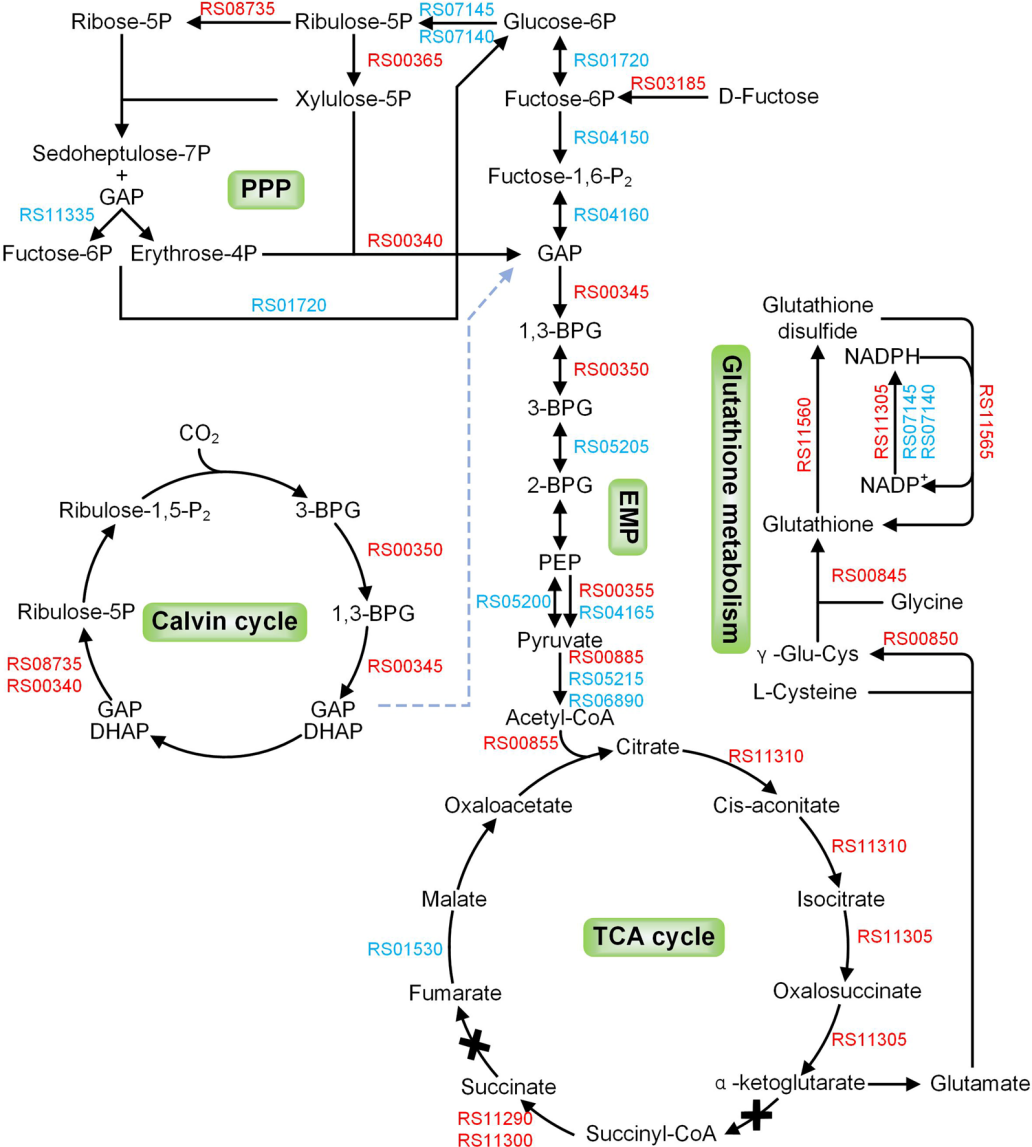
The gene expression differences in partial carbon metabolism pathways. Red and blue indicate up-regulated and down-regulated genes, respectively

Genes encoding carboxysome shell carbonic anhydrase, carboxysome peptide B and VWA domain-containing protein, which are associated with CO_2_ concentration, were up-regulated. In the Calvin cycle, the genes *pgk*, *gap*, *tkt* and *rpiA* were up-regulated. Up-regulation of genes associated with carbon fixation may help to provide precursors for other carbon metabolic pathways, such as glyceraldehyde 3-phosphate (GAP) in the EMP pathway. In the pentose phosphate pathway, one gene (*zwf*) involved in the oxidative phase was down-regulated and three genes (*rpe*, *tkt*, *rpiA*) involved in the non-oxidative phase were up-regulated. This suggests that *A. caldus* may require more ribose 5-phosphate for its involvement in nucleotide metabolism (e.g. DNA and RNA synthesis) under salt stress. The genes A5904_RS00345, A5904_RS00350, A5904_RS00355 encoding type I glyceraldehyde-3-phosphate dehydrogenase, phosphoglycerate kinase and pyruvate kinase in the glycolytic pathway were up-regulated. Genes responsible for the process from acetyl-CoA to α-ketoglutaric acid were upregulated in the tricarboxylic acid cycle. The accumulation of α-ketoglutaric acid contributes to the synthesis of glutamate, which provides the precursor for glutathione synthesis. Thus, the carbon flow appears to be towards glutathione synthesis, as shown in Fig. 7.

#### Energy metabolism

RISC oxidation involves electron transfer via the quinol pool (QH_2_). This can be a direct transfer to the terminal oxidases *bd* or *bo*_*3*_, resulting in a proton gradient and ATP generation, or a direct transfer to NADH complex I, generating reducing power [13]. The genes *cydB* and *cydA*, encoding cytochrome *bd* ubiquinol oxidase, and *cyoA*, encoding cytochrome *bo*_*3*_ ubiquinol oxidase, were down-regulated. Meanwhile, many genes encoding NADH complex I operon were down regulated, such as *nuoJ*, *nuoE*, *nuoB*, *nuoG*, *nuoA*, *nuoCD*, *nuoH*, *nuoI* and *nuoK*. The down-regulation of these genes indicates that high salt stress inhibits energy production, which leads to growth inhibition of the strain.

#### Amino acid transport and metabolism

Glutathione (GSH) is composed of three amino acids, glutamate, cysteine and glycine, which also act as GSH precursors. The genes *gshA* and *gshB*, encoding glutamate-cysteine ligase and glutathione synthase respectively, were up-regulated during GSH biosynthesis. GSH plays a significant part in the restoration of the redox balance of lipids and proteins in microorganisms. Specifically, GSH interacts both directly with proteins to reduce their disulfide bridges or indirectly via glutaredoxins that can then be reduced by GSH reductase with NAD(P)H as a cofactor [14]. In addition, the genes (A5904_RS11565 and A5904_RS11560) encoding two key enzymes (glutathione-disulfide reductase and glutathione peroxidase) of the glutathione system were up-regulated. The glutathione redox system can provide an external dithiol electron donor for ribonucleoside reductase. The genes (A5904_RS11765 and A5904_RS11760) encoding ribonucleotide reductases were upregulated. This enzyme catalyzes the rate-limiting de novo synthesis of 2′-deoxyribonucleic acid from ribonucleotides, which can provide the error-free deoxyribonucleic acid needed for DNA replication and repair [15]. In addition to their role in DNA replication and repair, glutathione reductase and glutathione peroxidase are both closely associated with resistance to oxidative stress in microorganisms.

In addition to glutathione metabolism, *argC*, a gene involved in arginine biosynthesis, was up-regulated. The gene (A5904_RS09360) encoding the glycine cleavage system protein H was down-regulated by 3.04-fold, probably to allow *A. caldus* to accumulate glycine at high chloride ion concentrations. The genes *pheA*, *aroC*, and *aroF* involved in the biosynthesis of phenylalanine, tyrosine, and tryptophan were up-regulated.

#### Cell motility

The genes associated with cell motility are mainly flagellar-associated genes. Down-regulation of the genes *flgB*, *flgL*, *flgE*, *flgS* and *fliC* encoding flagellar assembly proteins was observed. Furthermore, the genes *motD* and *motB* encoding flagellar motor proteins were downregulated. The downregulation of these genes implies a decrease in flagellar synthesis and rotation capacity. Flagellar synthesis requires sufficient energy, so down-regulation of related genes assists in the conservation of energy for other metabolic activities when exposed to salt stress. Furthermore, reduced flagellar synthesis also enhances both colonization and the formation of biofilm in the bacteria [16]. Thus, restriction of flagellar energy, such as that used for chemotaxis and motility, appears to favored under conditions of stress. It is worth noting that the down-regulation of flagellar-associated genes suggests that more active cells may be attached to sulfur particles, and thus a detergent can be used to release attached cells for future analysis.

#### Cell wall/membrane/envelope biogenesis

The cell wall of *A. caldus* consists of a thin layer of peptidoglycan and an outer membrane. High chloride levels negatively affect membrane integrity and therefore genes related to membrane components, such as lipopolysaccharides, phospholipids, outer membrane proteins and lipoproteins, are altered accordingly. Genes encoding penicillin-binding transpeptidase domain-containing proteins were up-regulated during peptidoglycan biosynthesis. In addition, two genes encoding GDP-mannose 4,6-dehydratase and NAD-dependent epimerase/dehydratase family protein were up-regulated, which contributes to the biosynthesis of the O-antigen. The genes encoding ABC transporter permease and ABC transporter ATP-binding protein, which are responsible for the O-antigen export system, were up-regulated. The gene encoding WbeA, which is located on the same operon as the above four genes, was also up-regulated. Genes involved in lipid A biosynthesis, *lpxC* and *lpxH*, were also up-regulated. O-antigen and lipid A are important components of lipopolysaccharide (LPS), and upregulation of related genes contributes to LPS biosynthesis. Meanwhile, *lptE*, a gene involved in LPS transport, was up-regulated. The upregulation of these genes suggests alterations of LPS assembly under high salt stress. The gene (A5904_RS10245) encoding the MlaC protein that can help restore lipid asymmetry of the outer membrane was up-regulated. In addition, the outer membrane protein A has an important part in the maintenance of membrane integrity and stability, and the periplasmic domain of the protein has been proposed to be involved in the tolerance of both osmotic and acid stress in *E. coli* [17]. Specifically, the genes encoding OmpA family proteins were down-regulated under salt stress. Reduced expression of these proteins leads to the presence of fewer pores in the outer membrane, which may prevent the entry of chloride into the cell. Increased production of periplasmic glucans during osmotic adaptation has also been reported in *Acidihalobacter* species [18]. Similarly, *mdoH* and *mdoG*, genes involved in glucan biosynthesis in *A. caldus*, were up-regulated under salt stress. Notably, both osmotic stress and altered gene expression under salt stress caused changes in cell morphology. Changes in genes related to re-structuring of cell wall and membrane may be mainly aimed at resisting cell shrinkage caused by salt stress. However, it is not certain which of the two is dominant in affecting cell morphology.

#### Information storage and processing

High levels of chloride ions can lead to DNA damage, necessitating the activation of repair mechanisms. DEGs classified as “nucleotide transport and metabolism” are mainly associated with purine and pyrimidine metabolism. Most of the DEGs involved in these two pathways were up-regulated, such as *purD*, *rdgB*, *pyrR*, *nrdB*, *nrdA*, *pyrB*, *pyrD*, *purH*, and *purB*. At the same time, numerous genes associated with DNA replication, recombination, and repair were up-regulated. For example, genes involved in mismatch repair such as *mutS2*, *dnaQ*, *dnaN* and *uvrD*, genes involved in base excision repair such as *nth*, and genes involved in homologous recombination such as *recF* and *recC*. The gene encoding a DUF4160 domain-containing protein located on the same operon as the gene encoding site-specific integrase was up-regulated by 18.285-fold. In addition, previous studies have shown that high gene turnover is associated with evolutionary adaptations in *A.caldus*, and high levels of genetic exchange have been observed in species inhabiting harsh environments [19]. This flexibility in the genetic repertoire provides the raw material for adaptation, specifically in terms of diversification of both phenotype and function. Our study also observed up-regulation of many genes encoding transposase that under high salt stress, including transposases belonging to the IS66 and IS5 families. The gene (A5904_RS02815) encoding the putative protein located on the same operon as the IS66 family transposase was significantly up-regulated (13166.662-fold), so we speculate that its function is related to transposition.

Bacteria can respond to environmental stresses through multiple patterns of gene transcriptional regulation. For example, various regulatory elements, such as transcriptional regulators and σ-factors, can regulate gene expression. The gene encoding the heat-inducible transcriptional repressor HrcA was up-regulated, consistent with the upregulation of heat shock proteins (e.g. groEL and groES). Genes encoding LysR family transcriptional regulators (A5904_RS07300 and A5904_RS04180) were up-regulated. The products of these two genes are associated with a variety of processes, including metabolic pathways, cell division, virulence, quorum sensing, motility, fixation of nitrogen, the response to oxidative stress, toxin synthesis, attachment, and secretion [20]. The gene (A5904_RS11725) encoding MerR family transcriptional regulators known to be involved in defense against oxidative and heavy-metal stress was up-regulated. The gene (A5904_RS00600) encoding a MarR family EPS-associated transcriptional regulator was also up-regulated under high salt stress. The sigma-70 family RNA polymerase sigma factor is a house-keeping protein that is associated with the high growth efficiency of bacteria [21]. The corresponding gene (A5904_RS02335) was down-regulated, consistent with the observed biomass reduction under high-salt stress.

As an important player in protein homeostasis, chaperone proteins assist in the assembly and folding of nascent peptides by recognizing and stabilizing the partially folded conformation of the peptide [22]. Under high-salt conditions, the genes *hscA*, *hscB*, *groEL* and *groES* encoding chaperone proteins were up-regulated, thus helping to maintain the proper folding of the peptide. Two genes (A5904_RS02240 and A5904_RS09190) encoding glutathione S-transferase were up-regulated. This enzyme catalyzes the glutathionylation of sulfhydryl proteins, resulting in reversible inactivation and protection from oxidative damage [23].

#### Defense mechanisms

The gene (A5904_RS11470) encoding the ABC transporter ATP-binding protein was upregulated 13.561-fold. These transporter proteins may play a role in nutrient uptake and toxin secretion, thus contributing to the survival of the strain under high salinity conditions. ROS interactions with organic molecules leads to the production of peroxides that cause damage to both membranes and proteins. Accordingly, *ahpC*, which encodes a peroxiredoxin subunit involved in the scavenging of organic peroxides, was upregulated. The HlyD family secretion proteins belong to the type I secretion system, which secretes substances such as adhesins involved in biofilm formation, MARTX, lipases and proteases, S-layer proteins, bacteriocin, and iron-scavenging proteins [24]. Therefore, up-regulation of the gene (A5904_RS12185) encoding the Hly protein may contribute to biofilm formation. The gene encoding NUDIX hydrolase was upregulated 2.726-fold. This enzyme can catalyze various important molecules, including, signaling molecules, intermediates, and coenzymes, that are potentially toxic. Thus, this enzyme is predicted to have the function of the removal of toxic metabolites and maintaining normal cellular homeostasis [25].

#### qRT-PCR verification of some DEGs

**Fig. 8:**
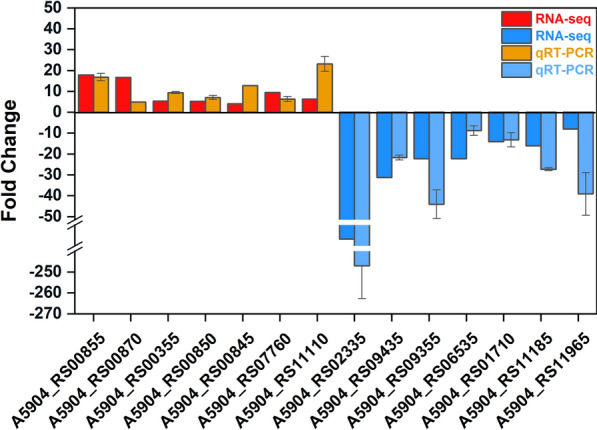
qRT-PCR verification of transcriptome data

As shown in Fig. 8, 14 DEGs were randomly chosen for qRT-PCR verification of the transcriptome results, and the results showed that seven up-regulated genes (A5904_RS00855, RS00870, RS00355, RS00850, RS00845, RS07760, RS11110) and seven down-regulated genes (A5904_ RS02335, RS09435, RS09355, RS06535, RS01710, RS11185, RS11965) showed the same up- and down-regulation trends in both sets of experiments, which indicating that the transcriptomic data were reliable.

### Effects of compatible solutes on the physiological properties of ***A. caldus***

#### Effects of the addition of exogenous compatible solutes on the physiological properties of *A. caldus*

**Fig. 9:**
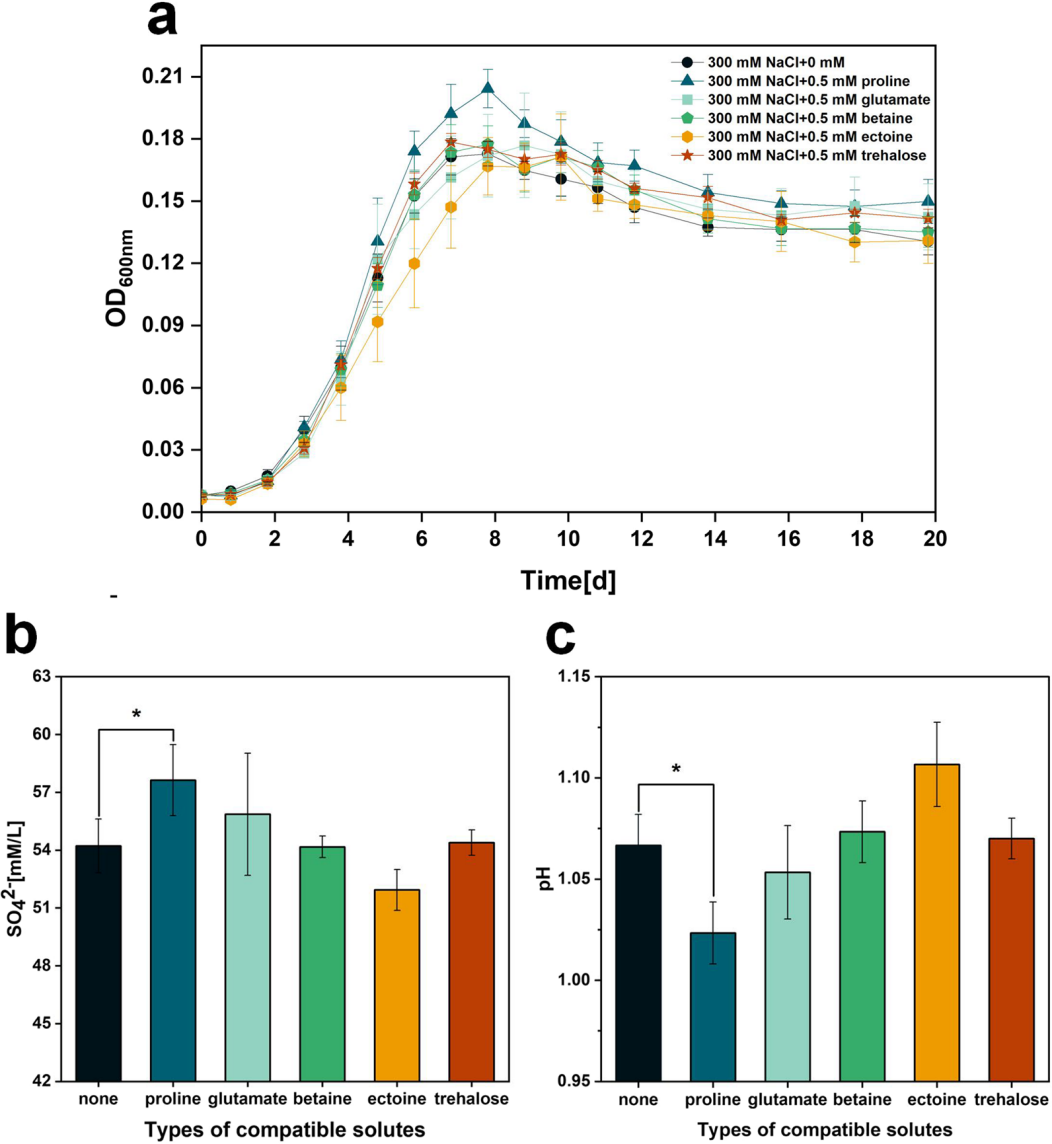
**a** OD_600_, **b** final SO_4_^2−^ content and **c** final pH of culture broth of *A. caldus* after exogenous addition of five compatible solutes. P values were assessed by t tests. * P < 0.05

The strains were cultured in Starkey-S^0^ liquid medium containing 300 mM NaCl together with 0.5 mM proline, glutamate, betaine, ectoine or trehalose, respectively. The effects of these five compatible solutes on the tolerance of the strains under NaCl stress were characterized by growth curves, SO_4_^2−^ content and pH. As shown in Fig. 9a, the strain with exogenous addition of proline had a significant growth advantage compared to the control without exogenous addition. As shown in Fig. 9b, the final SO_4_^2−^ content of the culture broth was 3.41 mM/L higher than the control after exogenous addition of proline. Correspondingly, Fig. 9c shows that the final pH of the culture broth was lowest after exogenous addition of proline. Taken together, proline is the most effective compatible solute in *A. caldus* compared to the other four compatible solutes. This may be because *A. caldus* has the De novo synthesis pathway of proline using glutamate as a precursor. It is noteworthy that the exogenous addition of glutamate is not as effective as proline, suggesting that the strain prefers to utilize proline under salt stress. The concentration of compatible solutes used above is referenced from the literature [26], so the optimum concentration may require further experimentation to determine.

#### Physiological properties of strains overexpressing proline biosynthesis genes

**Fig. 10:**
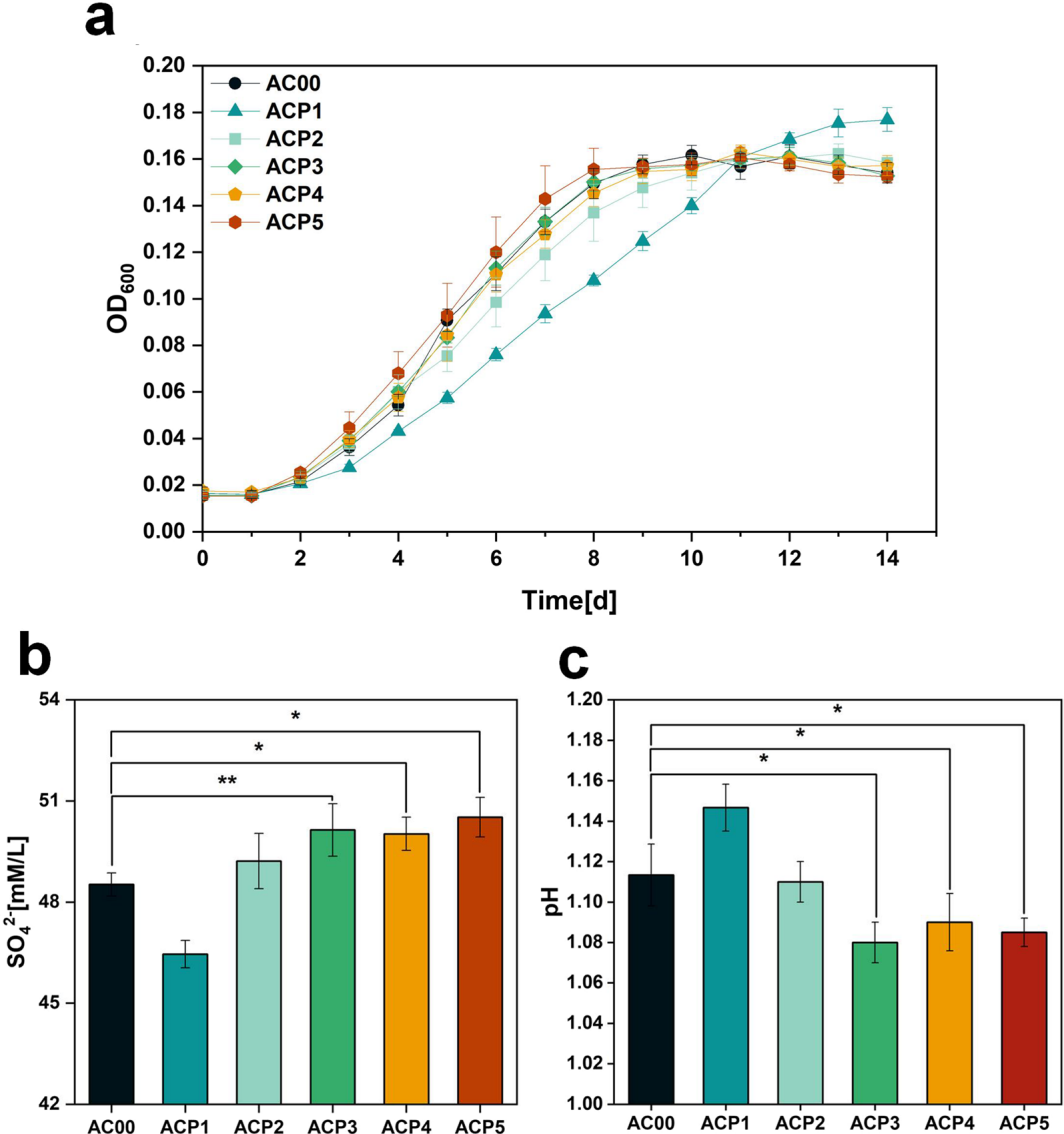
**a** OD_600_, **b** final SO_4_^2−^ content and **c** final pH of the culture broth of strains AC00, ACP1, ACP2, ACP3, ACP4 and ACP5. P values were determined by t tests. *P < 0.05, **P < 0.01

Strains AC00, ACP1, ACP2, ACP3, ACP4 and ACP5 were cultured in Starkey-S^0^ liquid medium containing 300 mM NaCl. As shown in Fig. 10a, from d 2 to d 4, the OD_600_ of all strains except ACP1 was higher than that of AC00. From day 5, the OD_600_ of ACP2, ACP3, and ACP4 were basically indistinguishable from AC00, and the OD600 of ACP5 remained higher than that of AC00. During the stabilization period, there was no significant difference between the strains except for ACP1. Throughout the growth process, the growth of ACP1 was always slower than the other strains, and it was still in the logarithmic phase when the other strains had reached the stable phase. Overall, strains overexpressing proline biosynthesis genes did not have significant advantages over the control strain AC00 throughout the entire growth process. As shown in Fig. 10b, the final SO_4_^2−^ content of ACP3, ACP4 and ACP5 groups was higher than that of the control group AC00. Specifically, the ACP3, ACP4 and ACP5 groups were 1.61, 1.50 and 1.99 mM/L higher than the AC00 group, respectively. Notably, the final SO_4_^2−^ content of ACP5 group is higher than that of ACP4, probably because other genes other than *proC* in the *yggS-proC-yggT-DUF167* operon were also at play. As the YggT system has been reported to be involved in osmotic adaptation (e.g., involved in potassium ion transport), it may induce the adaptive advantage of greater tolerance to chloride in *A. caldus* [27]. Although the specific involvement of YggS in osmotic regulation has not yet been experimentally demonstrated, its widespread conservation suggests that it may function together with YggT [26]. Correspondingly, Fig. 10c shows that the final pH of all groups showed the same trend as the final SO_4_^2−^ content.

Therefore, overexpression of *proA* (ACP1) had the opposite effect and this could be due to the consumption or titration of NADP^+^ away from other cellular processes that are already strained under salt stress. Equally likely, its overexpression could impact arginine synthesis, which is supported by the clearly negative effect of its overexpression on cell growth. Overexpression of *proB2*, *proC*, and *yggS-proC-yggT-DUF167* has a certain effect on improving the sulfur oxidation ability of *A. caldus* under sodium chloride stress. However, the final SO_4_^2−^ content was not increased to a great extent. This could suggest that both *proB2* and *proC* are responsible for potentially rate-limiting steps in proline synthesis. At the same time, we did not detect intracellular proline content, which may be due to the fact that the content did not reach the detection limit. The low intracellular proline content may have contributed to the low degree of improvement of *A. caldus* in salt tolerance. Similarly, the proline content detected when *A. thiooxidans* was under osmotic stress (300, 500, 700, and 1000 mM MgSO_4_) was not high (~ 11 µg/mg protein), although this content was significantly higher than that of the control without added MgSO_4_ [28]. Low concentrations of this amino acid suggest that proline appears to play a role in osmoregulation not as a compatible solute but as a protective substance. In addition, co-overexpression of genes (*proB*, *proA* and *proC*) involved in endogenous proline biosynthesis in *E. coli* was shown to contribute to proline biosynthesis and accumulation [29]. Therefore, we speculate that simultaneous overexpression of the four genes responsible for proline biosynthesis as a single operon may further enhance the sulfur oxidation capacity of *A. caldus* under NaCl stress. The combinatorial optimization of these four genes and the identification of suitable regulatory elements (e.g. promoters and RBS) for these genes are also future considerations.

### Effects of glutathione on the physiological properties of ***A. caldus***

#### Effects of exogenous glutathione addition on the physiological properties of *A. caldus*

**Fig. 11:**
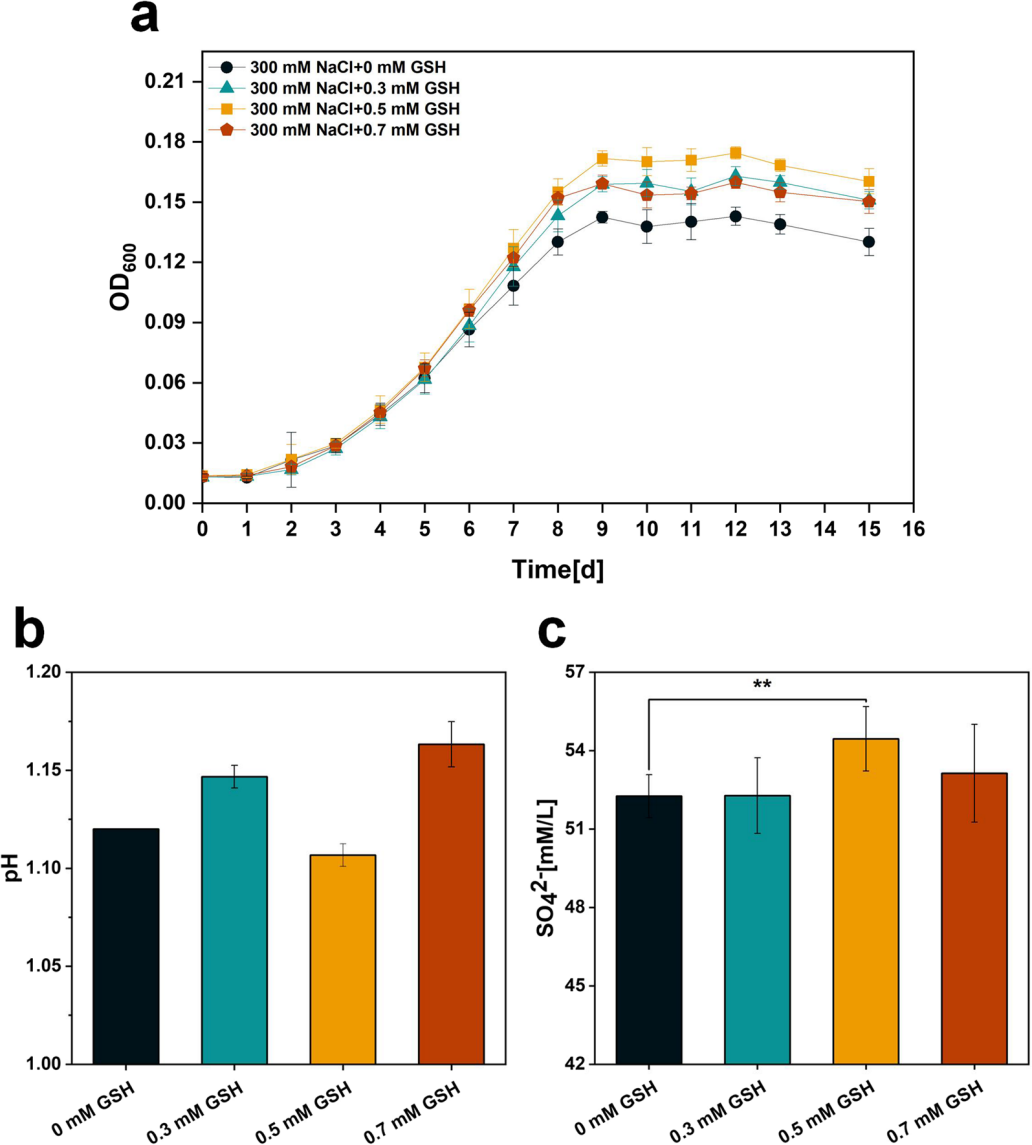
**a** OD_600_, **b** final SO_4_^2−^ content and **c** final pH of culture broth of *A. caldus* after exogenous addition of different GSH concentrations. P values were determined by t tests. **P < 0.01

The strains were cultured in Starkey-S^0^ liquid medium containing 300 mM NaCl plus 0, 0.3, 0.5, 0.7 mM glutathione, respectively. The effects of various glutathione concentrations on the tolerance of the strains under NaCl stress were characterized by growth curves, SO_4_^2−^ content and pH. As shown in Fig. 11a, the OD_600_ of the experimental groups with exogenous GSH addition was higher than the control group without GSH addition from the 6th day onwards. Among them, the strain showed a significant growth advantage when 0.5 mM GSH was added externally. Figure 11b shows that only the experimental group with 0.5 mM GSH added externally had a significantly higher SO_4_^2−^ content, 2.2 mM/L more than the control group. Similarly, Fig. 11c shows that the pH was the lowest in the experimental group with an exogenous addition of 0.5 mM GSH. This may be because the exogenous addition of GSH as an antioxidant will increase both glutathione peroxidase and catalase activities, to eliminate excessive reactive oxygen species (ROS) in cells resulting from oxidative stress [30].

#### Physiological properties of strains overexpressing glutathione biosynthesis genes

**Fig. 12:**
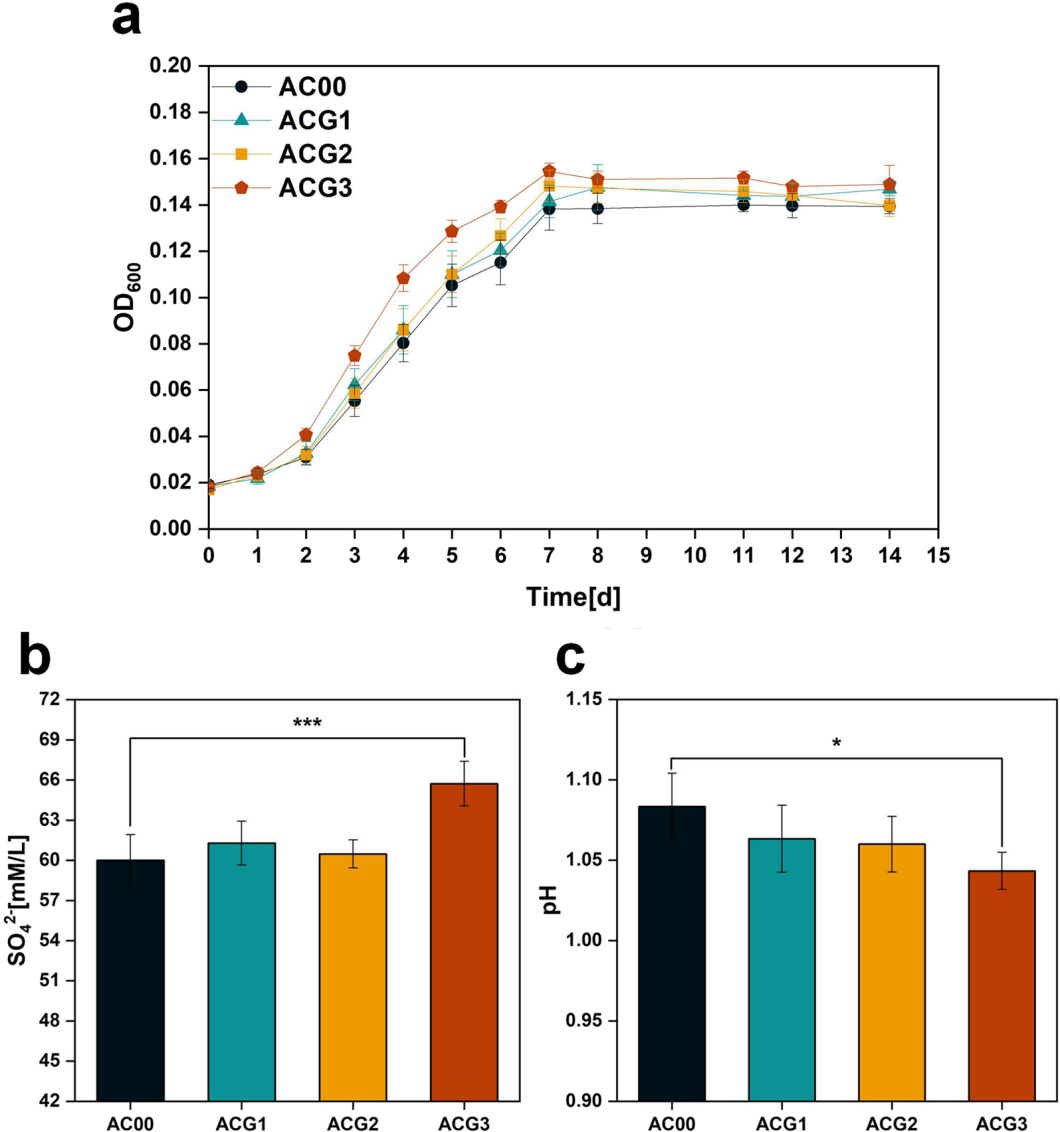
**a** OD_600_, **b** final SO_4_^2−^ content and **c** final pH of the culture broth of strains AC00, ACG1, ACG2 and ACG3. P values were determined by t tests. *P < 0.05, ***P < 0.001

Strains AC00, ACG1, ACG2 and ACG3 were cultured in Starkey-S^0^ liquid medium containing 300 mM NaCl. As shown in Fig. 12a, the OD_600_ of the ACG1, ACG2 and ACG3 groups were higher than that of the control group AC00 from day 3 until the stabilization period. Among them, the OD_600_ of the ACG3 group was more significantly higher than that of the AC00 group. As shown in Fig. 12b, the final SO_4_^2−^ content of ACG1, ACG2 and ACG3 groups was higher than that of the control group AC00. Specifically, the ACG1, ACG2 and ACG3 groups were 1.28, 0.47 and 5.72 mM/L higher than the AC00 group, respectively. As shown in Fig. 12c, the final pH of ACG1, ACG2 and ACG3 groups were lower than the control group AC00. The final pH of the ACG3 group was the lowest, following the same trend as the final SO_4_^2−^ content. Therefore, the growth ability and sulfur oxidation capacity of ACG3 were superior to other strains under NaCl stress. This suggests that simultaneous overexpression of *gshA* and *gshB* as a single operon helps *A. caldus* to better perform bioleaching operations in environments containing sodium chloride stress.

**Fig. 13:**
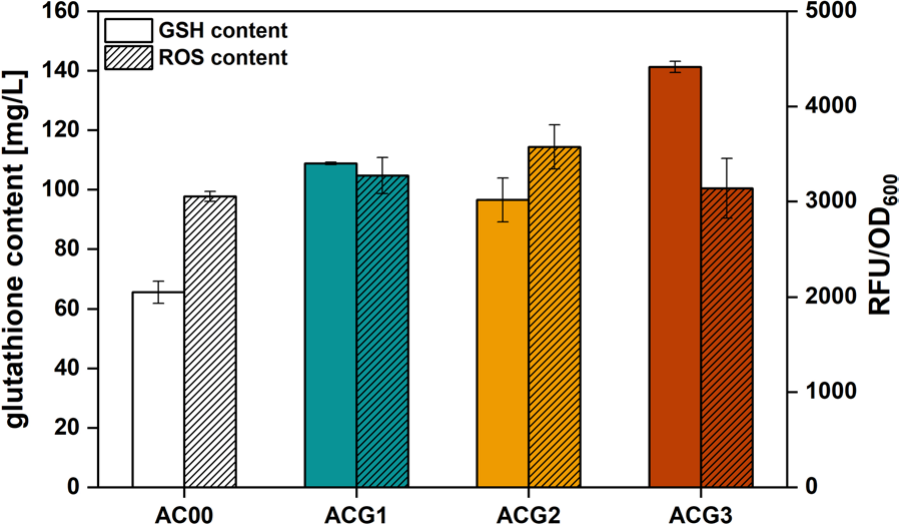
Intracellular glutathione and reactive oxygen species content of strains AC00, ACG1, ACG2 and ACG3.

Overexpression of genes responsible for glutathione biosynthesis genes would be expected to increase glutathione production in *A. caldus*. The measurement results showed that the intracellular glutathione content of ACG1, ACG2 and ACG3 was higher than that of AC00. Notably, ACG3 had the highest intracellular glutathione content, which should account for its adaptive advantage under NaCl stress. Previous studies have shown that glutathione is an effective scavenger of ROS, so we further measured the intracellular levels of ROS formed by respiration-induced. However, Fig. 13 shows that the intracellular ROS content of ACG1, ACG2 and ACG3 were higher than that of AC00. This suggests that rather than directly lowering the ROS concentrations, glutathione may mitigate damage to cellular macromolecules, even in the presence of excess ROS [30]. This also suggests that overexpression of glutathione biosynthesis genes allows for higher respiration rates, thereby pumping out excess protons and lowering oxygen levels to maintain pH homeostasis.

## Conclusion

Here, the physiological responses of *A. caldus* MHT-04 toward various NaCl conditions were investigated. The higher the concentration of NaCl, the more pronounced its inhibitory effect on *A. caldus*. In particular, *A. caldus* was completely inhibited at a concentration of 800 mM NaCl. Comparative transcriptomics was used to assess transcriptional differences in genes, followed by the verification of selected genes by RT-qPCR. The overall conclusions from the analysis of *A. caldus* MHT-04 adaptation to NaCl stress are as follows. Genes associated with the TCA cycle and glutathione metabolism are upregulated to synthesize more glutathione in response to oxidative stress. Reduced flagellar synthesis assists in reducing energy expenditure. Changes in cell wall and membrane composition in response to the effects of high chloride ion concentrations on membrane integrity. Damage to both DNA and protein produced by high chloride ion concentrations is repaired to protect processes such as transcription, protein synthesis, and enzymatic reactions. Some genes associated with defense mechanisms are also up-regulated in response to NaCl stress. In addition, we demonstrated that proline and glutathione contribute to the adaptation of *A. caldus* under NaCl stress by means of exogenous additions and genetic engineering modifications. In the proline- and glutathione-related experimental groups, simultaneous overexpression of *gshA* and *gshB* as an operon best facilitated sulfur oxidation in *A. caldus* under NaCl stress. The findings of this investigation provide novel insights into mechanisms underlying the adaptation of *A. caldus* to high-salt stress, as well as a foundation for the use of these acidophiles in industrial bioleaching processes.

### Supplementary Information

**Additional file 1: Text S1. Text S2. Fig. S1.** Colony PCR validation results for AC00, ACP1, ACP2, ACP3, ACP4 and ACP5. **Text S3. Fig. S2**. Colony PCR validation results for ACG1, ACG2 and ACG3. **Fig. S3**. The construction process of plasmids pJRD215-Ptac, pJRD215-Ptac-proA, pJRD215-Ptac-proB1, pJRD215-Ptac-proB2, pJRD215-Ptac-proC and pJRD215-Ptac- yggS-proC-yggT-DUF167. **Fig. S4**. The construction process of plasmids pJRD215-Ptac-gshA, pJRD215-Ptac-gshB and pJRD215-Ptac-gshAB. **Table S1**. Primers used in the qRT-PCR experiment. **Table S2**. Primers used for plasmid construction. **Table S3**. Details of differentially expressed genes.

## Data Availability

Not applicable.

## Abbreviations

RISCs: Reduced inorganic sulfur compounds
DEGs: Differentially expressed genes
GSH: Glutathione
ROS: Reactive oxygen species
LPS: Lipopolysaccharide
