# Supplementary material for: Study on the intracellular adaptative mechanism of Acidithiobacillus caldus MTH-04 to NaCl stress
Source: Microb Cell Fact. 2023 Oct 25;22:218. doi: 10.1186/s12934-023-02232-w (PMC10599003; doi:10.1186/s12934-023-02232-w)
Supplement: Supplementary file 1 — Additional file 1: Text S1. Text S2. Fig. S1. Colony PCR validation results for AC00, ACP1, ACP2, ACP3, ACP4 and ACP5. Text S3. Fig. S2. Colony PCR validation results for ACG1, ACG2 and ACG3. Fig. S3. The construction process of plasmids pJRD215-Ptac, pJRD215-Ptac-proA, pJRD215-Ptac-proB1, pJRD215-Ptac-proB2, pJRD215-Ptac-proC and pJRD215-Ptac- yggS-proC-yggT-DUF167. Fig. S4. The construction process of plasmids pJRD215-Ptac-gshA, pJRD215-Ptac-gshB and pJRD215-Ptac-gshAB. Table S1. Primers used in the qRT-PCR experiment. Table S2. Primers used for plasmid construction. Table S3. Details of differentially expressed genes. [file 12934_2023_2232_MOESM1_ESM.docx]

**Additional file**

**Text S1**

The culture broth was centrifuged at 12000 rpm for 3 min to remove the bottom sediment and the supernatant was diluted 50-fold. To 500 μl of the diluted supernatant, 40 μL of 2.5 M hydrochloric acid solution and 100 μL of 1 M BaCrO_4_ suspension were added. The above suspension was mixed well and then reacted in a shaker at 40°C for 10 min. Then 80 μL of ammonia was added to the suspension. After mixing and standing for 5 min, the suspension was centrifuged at 12000 rpm for 3 min to obtain the supernatant. 200 μL of supernatant was added to a 96-well plate and detected at 420 nm with a microplate reader.

**Text S2**

When colony PCR was used to validate AC00, ACP1, ACP2, ACP3, ACP4 and ACP5, their theoretical band sizes were 366, 1491, 1483, 1499, 1194 and 2553 bp, respectively. As shown in Fig. S1, the positions of the bands in lanes 1-4, 5-8, 9-11, 13-16, 17-20, and 21-24 indicate the successful construction of the AC00, ACP1, ACP2, ACP3, ACP4, and ACP5 strains, respectively.


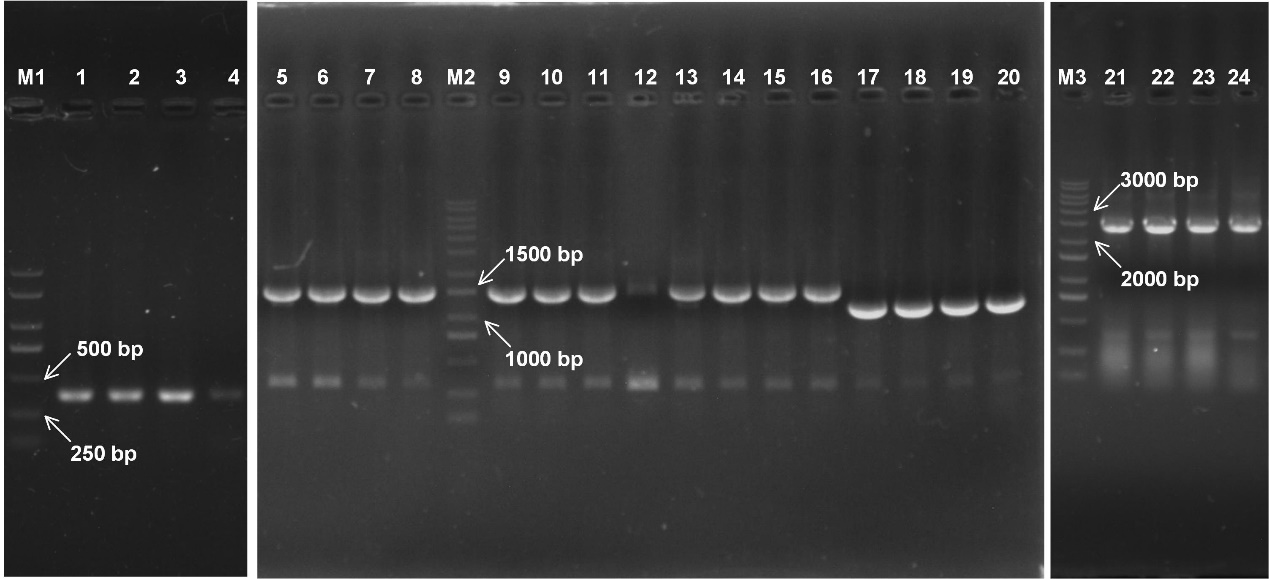


**Fig. S1** Colony PCR validation results for AC00, ACP1, ACP2, ACP3, ACP4 and ACP5. Lanes 1-4: AC00, lanes 5-8: ACP1, lanes 9-12: ACP2, lanes 13-16: ACP3, lanes 17-20: ACP4, lanes 21-24: ACP5, lane M1:BM2000 DNA Ladder, lanes M2 and M3:1 kb Plus DNA Ladder.

**Text S3**

When colony PCR was used to validate ACG1, ACG2 and ACG3, their theoretical band sizes were 1,662, 1,295, and 2,587 bp, respectively. As shown in Fig. S2, the positions of the bands in lanes 1-4 indicate the successful construction of the ACG1 strain, the positions of the bands in lanes 5 and 6 indicate the successful construction of the ACG2 strain, and the positions of the bands in lanes 9, 11, and 12 indicate the successful construction of the ACG3 strain.


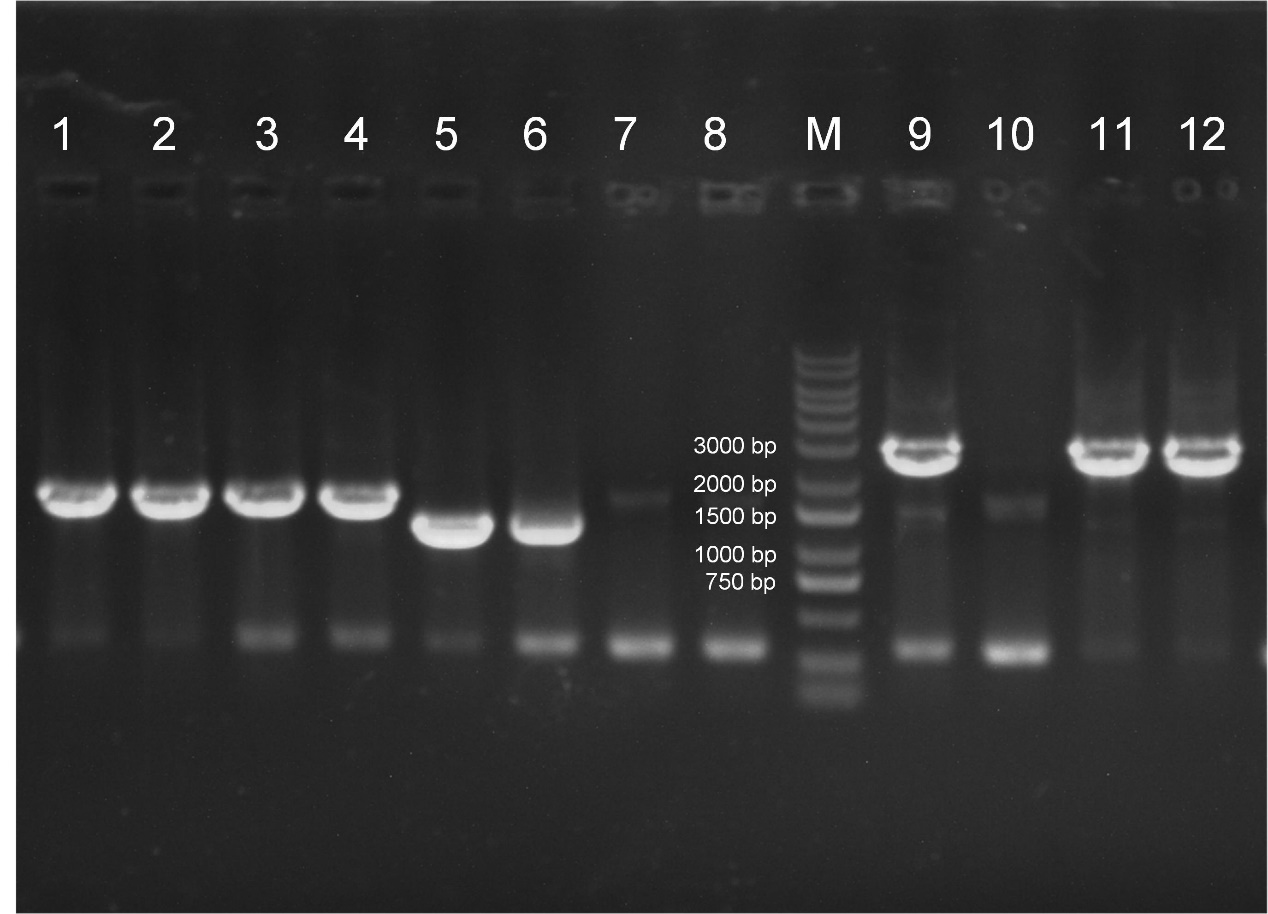


**Fig. S2** Colony PCR validation results for ACG1, ACG2 and ACG3. Lanes 1-4: ACG1, lanes 5-8: ACG2, lanes 9-12: ACG3, lane M: 1 kb Plus DNA Ladder.


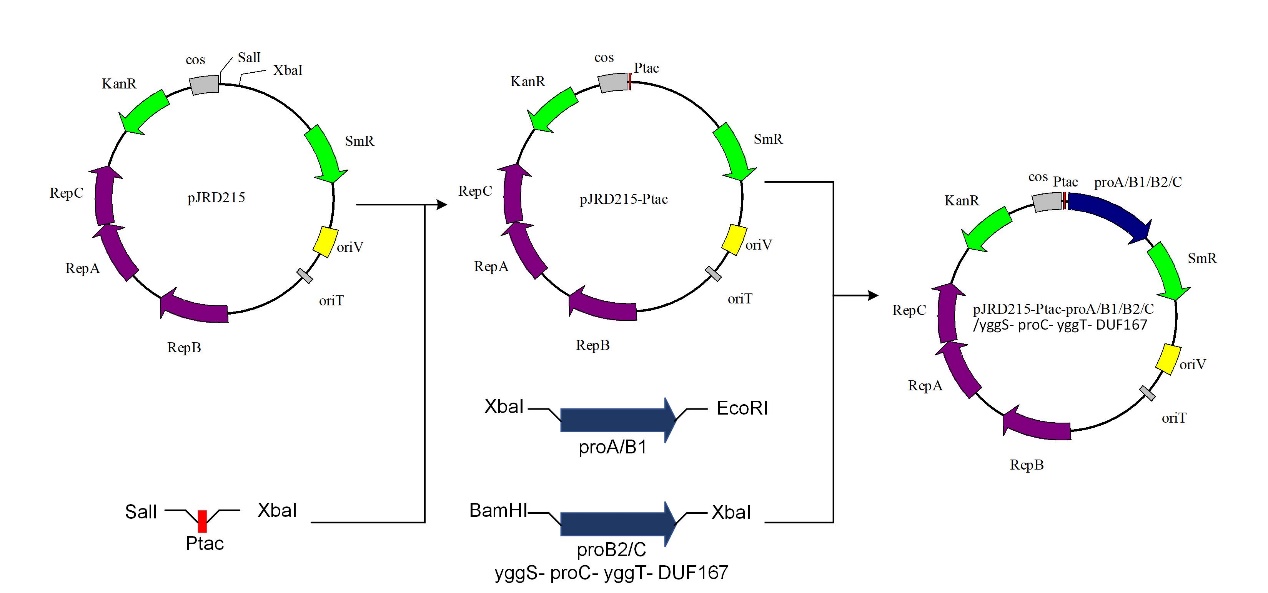


**Fig. S3** The construction process of plasmids pJRD215-Ptac, pJRD215-Ptac-proA, pJRD215-Ptac-proB1, pJRD215-Ptac-proB2, pJRD215-Ptac-proC and pJRD215-Ptac- yggS-proC-yggT-DUF167.


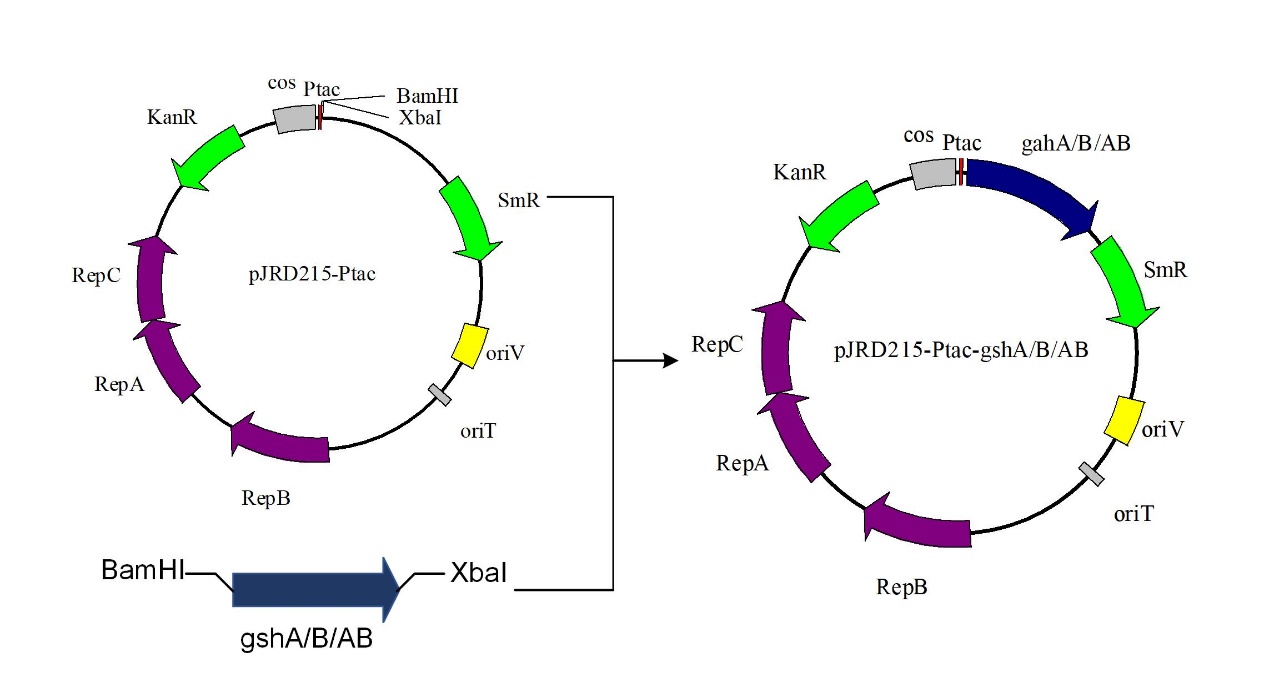


**Fig. S4** The construction process of plasmids pJRD215-Ptac-gshA, pJRD215-Ptac-gshB and pJRD215-Ptac-gshAB.

**Table S1** Primers used in the qRT-PCR experiment

| Name | Description | Sequence (5’-3’) |
| --- | --- | --- |
| 03910-F | type I methionyl aminopeptidase | GAACATCTTGCTGCTGTCGCC |
| 03910-R |  | TCTGCATTTCCCTCAATCACG |
| 00855-F | citrate synthase | ATCAACGCCAGCACCTTCTC |
| 00855-R |  | TCGATGACCTTCTGGTTGGC |
| 00870-F | ferrous iron transport protein B | TTTGTCCTGCTCTACCTGCC |
| 00870-R |  | CGTCGCAAGACCGTAGAAGA |
| 00355-F | pyruvate kinase | TTCCGAAGTGGTCATGGTGG |
| 00355-R |  | ATCTGTGTTGCGGTGATGGT |
| 00850-F | glutamate--cysteine ligase | CGGGCTTCAACAACCTCAAC |
| 00850-R |  | CGGATGGTATTGGGCAAGGT |
| 00845-F | glutathione synthase | GGAATACAACCCGCCAAGGA |
| 00845-R |  | GAGTTCGGCCAGGGTAAACA |
| 07760-F | Fe-S protein assembly chaperone HscA | GGAAGCGATGCCGATACCAT |
| 07760-R |  | ACGGTTCATACGCCTTTCCA |
| 11110-F | peroxiredoxin | AACCCGATGGAAGTAAGCCC |
| 11110-R |  | TAGTCCTTGAGTTGCCGCTG |
| 02335-F | sigma-70 family RNA polymerase sigma factor | GCGCAGAGGGTGTTATTTCG |
| 02335-R |  | AGCAGAGCTTCACTCGTCTC |
| 09435-F | FAD-dependent oxidoreductase | ACGTCTCCAGCGTTTCTACC |
| 09435-R |  | TGCTGCTCTGGAATGTCTGG |
| 09355-F | hemerythrin domain-containing protein | ATCGCATGTTGGAAGACGCA |
| 09355-R |  | CAGACTTTCTTCCACCGCCA |
| 06535-F | 2-hydroxyacid dehydrogenase | CTCATCGACACCAAGGCACT |
| 06535-R |  | GATGTCGGCTTCTTCCTCGT |
| 01710-F | cytochrome d ubiquinol oxidase subunit II | TGACCCTCTCCTTCGCCTAT |
| 01710-R |  | CAGGTGTTGGGCACGATGTA |
| 11185-F | MFS transporter | AGCAGAACTCCCACCACAAG |
| 11185-R |  | CGTAGAGCGGCAGAAATCCA |
| 11965-F | NADH-quinone oxidoreductase subunit A | CCTTTGAGAGTGGGCTTGGT |
| 11965-R |  | AGAAAGGACACCGCCAACAG |

**Table S2** Primers used for plasmid construction

| Name | Sequence (5’-3’) |
| --- | --- |
| 215MCS-F | aatcagcgacactgaatacgg |
| 215MCS-R | ttcatacacggtgcctgac |
| Ptac-F | tcgacttgacaattaatcatcggctcgtataatgggatccggtacct |
| Ptac-R | ctagaggtaccggatcccattatacgagccgatgattaattgtcaag |
| proA-F | gctctagacctcgccactagctaggagaccgtatg |
| proA-R | cggaattctcaggaacggatatggccgtcc |
| proB1-F | gctctagaccatgaggcagcgccagcac |
| proB1-R | cggaattcaccaatccctactccgtcgcgc |
| proB2-F | cgggatccgaaccatgaacggaccgc |
| proB2-R | gctctagattactggctgcgtgttgcggttag |
| proC-F | cgggatccggtgagcgaaccgcgaataattttc |
| proC-R | gctctagagatcattggttatgctcctcggtc |
| SCT167-F | ggctcgtataatgggatccagccgtcccagacattatgctg |
| SCT167-R | gtcaaaacaaagactctagaatcaggtcgttgaagggcagtt |
| gshA-F | cgggatccaacccatgatcggcgtaatggg |
| gshA-R | gtctagactcaagcacggctctgggtact |
| gshB-F | cgcggatcccgtgcttgaggctgccgt |
| gshB-R | gtctagagtcaggccccttgctccag |

**Table S3** Details of differentially expressed genes

| Gene_id | Gene description | Log_2_(Fold Change) | Padjust |
| --- | --- | --- | --- |
| **C: Energy production and conversion** | | | |
| **UP** |  |  |  |
| A5904_RS00855 | citrate synthase | 4.160 | 1.01E-07 |
| A5904_RS07755 | ISC system 2Fe-2S type ferredoxin | 3.314 | 1.46E-05 |
| A5904_RS07610 | NADH-dependent flavin oxidoreductase | 3.568 | 6.17E-05 |
| A5904_RS04270 | DUF3683 domain-containing protein | 3.438 | 7.43E-05 |
| A5904_RS07060 | class I SAM-dependent methyltransferase | 3.005 | 0.000428 |
| A5904_RS10890 | 4Fe-4S dicluster domain-containing protein | 1.661 | 0.004467 |
| A5904_RS11300 | ADP-forming succinate--CoA ligase subunit beta | 1.829 | 0.004561 |
| A5904_RS08480 | iron-containing alcohol dehydrogenase | 2.839 | 0.005522 |
| A5904_RS00370 | phosphoglycolate phosphatase | 1.706 | 0.006800 |
| A5904_RS06990 | NADH:flavin oxidoreductase/NADH oxidase | 1.915 | 0.007972 |
| A5904_RS00885 | FAD-dependent oxidoreductase | 1.524 | 0.007972 |
| A5904_RS11565 | glutathione-disulfide reductase | 1.666 | 0.009988 |
| A5904_RS11310 | aconitate hydratase | 2.026 | 0.010575 |
| A5904_RS08250 | NAD(P)-dependent glycerol-3-phosphate dehydrogenase | 1.969 | 0.015758 |
| A5904_RS03415 | 4Fe-4S dicluster domain-containing protein | 1.814 | 0.015769 |
| A5904_RS00045 | ferredoxin family protein | 2.254 | 0.016329 |
| A5904_RS10840 | 4Fe-4S dicluster domain-containing protein | 2.366 | 0.017075 |
| A5904_RS03395 | cytochrome ubiquinol oxidase subunit I | 1.915 | 0.020515 |
| A5904_RS10805 | NAD(P)/FAD-dependent oxidoreductase | 1.606 | 0.020803 |
| A5904_RS13570 | nitroreductase family protein | 2.028 | 0.030040 |
| A5904_RS11305 | NADP-dependent isocitrate dehydrogenase | 1.613 | 0.030897 |
| A5904_RS11290 | succinate--CoA ligase subunit alpha | 1.477 | 0.031190 |
| A5904_RS03420 | dimethyl sulfoxide reductase anchor subunit | 1.325 | 0.039951 |
| A5904_RS11820 | NADH-quinone oxidoreductase subunit D | 1.402 | 0.044815 |
| **DOWN** |  |  |  |
| A5904_RS09435 | FAD-dependent oxidoreductase | -4.982 | 1.65E-11 |
| A5904_RS01710 | cytochrome d ubiquinol oxidase subunit II | -3.819 | 2.59E-08 |
| A5904_RS10020 | cytochrome ubiquinol oxidase subunit I | -3.989 | 1.91E-07 |
| A5904_RS06535 | 2-hydroxyacid dehydrogenase | -4.470 | 6.91E-07 |
| A5904_RS04145 | bifunctional enoyl-CoA hydratase/phosphate acetyltransferase | -3.303 | 5.80E-05 |
| A5904_RS12005 | NADH-quinone oxidoreductase subunit J | -2.747 | 5.85E-05 |
| A5904_RS06890 | dihydrolipoyl dehydrogenase | -2.894 | 9.14E-05 |
| A5904_RS11980 | NADH-quinone oxidoreductase subunit E | -2.402 | 0.000142 |
| A5904_RS06895 | polyphosphate kinase 2 | -2.691 | 0.000163 |
| A5904_RS09990 | HAD family hydrolase | -3.098 | 0.001034 |
| A5904_RS11970 | NADH-quinone oxidoreductase subunit B | -2.965 | 0.001615 |
| A5904_RS01900 | SDR family oxidoreductase | -2.189 | 0.002362 |
| A5904_RS11210 | DsrE/DsrF/DrsH-like family protein | -2.568 | 0.002481 |
| A5904_RS03680 | FdhF/YdeP family oxidoreductase | -2.892 | 0.002666 |
| A5904_RS11990 | 2Fe-2S iron-sulfur cluster-binding protein | -2.349 | 0.004342 |
| A5904_RS09400 | nitrate reductase subunit alpha | -2.726 | 0.004655 |
| A5904_RS06505 | NAD(P)H:quinone oxidoreductase | -2.002 | 0.005298 |
| A5904_RS11965 | NADH-quinone oxidoreductase subunit A | -3.009 | 0.005592 |
| A5904_RS01530 | class II fumarate hydratase | -2.114 | 0.007091 |
| novel0002 | F0F1 ATP synthase subunit beta [Acidithiobacillus caldus] | -2.883 | 0.009281 |
| A5904_RS03970 | FAD/NAD(P)-binding oxidoreductase | -2.199 | 0.010575 |
| A5904_RS11975 | NADH-quinone oxidoreductase subunit D | -1.921 | 0.011891 |
| A5904_RS11995 | NADH-quinone oxidoreductase subunit NuoH | -1.964 | 0.013275 |
| A5904_RS07550 | rubrerythrin family protein | -2.415 | 0.015818 |
| A5904_RS12000 | NADH-quinone oxidoreductase subunit NuoI | -1.976 | 0.016518 |
| A5904_RS07565 | SDR family NAD(P)-dependent oxidoreductase | -1.670 | 0.017648 |
| novel0162 | rubrerythrin family protein [Acidithiobacillus caldus] | -2.782 | 0.018977 |
| A5904_RS09410 | nitrate reductase molybdenum cofactor assembly chaperone | -2.129 | 0.023207 |
| A5904_RS11165 | FAD-binding protein | -1.914 | 0.025598 |
| A5904_RS04155 | FAD-dependent oxidoreductase | -2.157 | 0.027074 |
| A5904_RS12010 | NADH-quinone oxidoreductase subunit K | -1.783 | 0.029028 |
| A5904_RS09405 | nitrate reductase subunit beta | -2.175 | 0.029165 |
| A5904_RS03685 | formate dehydrogenase accessory sulfurtransferase FdhD | -1.701 | 0.030860 |
| novel0133 | hypothetical protein [Acidithiobacillus caldus] | -4.711 | 0.032825 |
| A5904_RS10205 | ubiquinol oxidase subunit II | -2.139 | 0.042009 |
| novel0253 | FAD-dependent oxidoreductase, partial [Acidithiobacillus caldus] | -2.283 | 0.042803 |
| A5904_RS05215 | thiamine pyrophosphate-dependent enzyme | -1.659 | 0.042830 |
| A5904_RS08350 | cytochrome ubiquinol oxidase subunit I | -2.302 | 0.044901 |
| novel0078 | molybdopterin-dependent oxidoreductase [Acidithiobacillus caldus] | -2.917 | 0.046956 |
| **D: Cell cycle control, cell division, chromosome partitioning** | | | |
| **UP** |  |  |  |
| A5904_RS03225 | translesion DNA synthesis-associated protein ImuA | 1.738 | 0.018977 |
| A5904_RS02600 | septum formation initiator family protein | 1.642 | 0.021733 |
| A5904_RS08540 | penicillin-binding transpeptidase domain-containing protein | 1.460 | 0.022632 |
| **DOWN** |  |  |  |
| novel0184 | penicillin-binding protein 2 [Acidithiobacillus caldus] | -3.519 | 5.04E-06 |
| A5904_RS12480 | peptidoglycan DD-metalloendopeptidase family protein | -2.922 | 0.001309 |
| A5904_RS15020 | ParA family protein | -2.424 | 0.002155 |
| A5904_RS10970 | penicillin-binding protein 2 | -1.946 | 0.002311 |
| A5904_RS10975 | rod shape-determining protein RodA | -2.259 | 0.003260 |
| A5904_RS12705 | ribosome biogenesis GTP-binding protein YihA/YsxC | -2.343 | 0.005619 |
| **E: Amino acid transport and metabolism** | | | |
| **UP** |  |  |  |
| A5904_RS04280 | class II aldolase/adducin family protein | 2.478 | 0.000277 |
| A5904_RS04885 | prephenate dehydratase | 2.535 | 0.000855 |
| A5904_RS00845 | glutathione synthase | 2.016 | 0.002568 |
| A5904_RS09835 | chorismate synthase | 2.441 | 0.002808 |
| A5904_RS01690 | N-acetyl-gamma-glutamyl-phosphate reductase | 1.690 | 0.005143 |
| A5904_RS07885 | agmatine deiminase family protein | 1.833 | 0.011694 |
| A5904_RS12290 | PatB family C-S lyase | 1.870 | 0.011758 |
| A5904_RS01165 | methylenetetrahydrofolate reductase [NAD(P)H] | 1.499 | 0.015958 |
| A5904_RS11115 | 2-isopropylmalate synthase | 1.242 | 0.025005 |
| A5904_RS04880 | histidinol-phosphate transaminase | 2.084 | 0.037671 |
| A5904_RS04875 | 3-deoxy-7-phosphoheptulonate synthase | 1.409 | 0.040483 |
| A5904_RS05185 | succinyl-diaminopimelate desuccinylase | 1.255 | 0.041879 |
| A5904_RS07835 | dihydroxy-acid dehydratase | 1.267 | 0.047506 |
| **DOWN** |  |  |  |
| A5904_RS09360 | glycine cleavage system protein H | -3.042 | 8.65E-06 |
| A5904_RS09895 | O-succinylhomoserine sulfhydrylase | -2.472 | 0.000206 |
| A5904_RS03955 | FAD-binding oxidoreductase | -2.513 | 0.000419 |
| **F: Nucleotide transport and metabolism** | | | |
| **UP** |  |  |  |
| A5904_RS08510 | phosphoribosylamine--glycine ligase | 2.277 | 0.000278 |
| A5904_RS10450 | RdgB/HAM1 family non-canonical purine NTP pyrophosphatase | 1.931 | 0.001059 |
| A5904_RS00830 | bifunctional pyr operon transcriptional regulator/uracil phosphoribosyltransferase PyrR | 2.062 | 0.001754 |
| A5904_RS11760 | ribonucleotide-diphosphate reductase subunit beta | 2.605 | 0.004804 |
| A5904_RS13590 | HIT family protein | 1.877 | 0.006017 |
| A5904_RS11765 | ribonucleoside-diphosphate reductase subunit alpha | 2.321 | 0.012979 |
| A5904_RS00825 | aspartate carbamoyltransferase catalytic subunit | 1.940 | 0.021733 |
| A5904_RS08525 | quinone-dependent dihydroorotate dehydrogenase | 1.605 | 0.024528 |
| A5904_RS08505 | bifunctional phosphoribosylaminoimidazolecarboxamide formyltransferase/IMP cyclohydrolase | 1.665 | 0.027838 |
| A5904_RS09490 | type 1 glutamine amidotransferase | 1.789 | 0.031797 |
| A5904_RS10090 | adenylosuccinate lyase | 1.355 | 0.040483 |
| **DOWN** |  |  |  |
| A5904_RS15595 | DUF1971 domain-containing protein | -2.278 | 0.002847 |
| A5904_RS05330 | NAD(P)H-hydrate dehydratase | -1.611 | 0.021987 |
| A5904_RS02935 | FAD-dependent thymidylate synthase | -1.461 | 0.036207 |
| **G: Carbohydrate transport and metabolism** | | | |
| **UP** |  |  |  |
| A5904_RS03185 | PfkB family carbohydrate kinase | 3.030 | 1.67E-05 |
| A5904_RS00355 | pyruvate kinase | 2.423 | 0.000207 |
| A5904_RS00345 | type I glyceraldehyde-3-phosphate dehydrogenase | 2.351 | 0.000594 |
| A5904_RS00365 | ribulose-phosphate 3-epimerase | 2.110 | 0.000655 |
| A5904_RS00595 | ABC transporter permease | 2.953 | 0.000933 |
| A5904_RS00340 | transketolase | 2.190 | 0.001615 |
| A5904_RS00350 | phosphoglycerate kinase | 1.844 | 0.010665 |
| A5904_RS08735 | ribose-5-phosphate isomerase RpiA | 1.987 | 0.011850 |
| A5904_RS00590 | ABC transporter ATP-binding protein | 2.163 | 0.013633 |
| A5904_RS01145 | carbohydrate kinase family protein | 1.469 | 0.021225 |
| A5904_RS01195 | MFS transporter | 1.713 | 0.027581 |
| A5904_RS09830 | MFS transporter | 2.268 | 0.043783 |
| **DOWN** |  |  |  |
| A5904_RS10370 | glycogen debranching protein GlgX | -4.074 | 1.25E-09 |
| A5904_RS12745 | sugar porter family MFS transporter | -3.321 | 7.55E-07 |
| A5904_RS11185 | MFS transporter | -4.001 | 5.11E-06 |
| A5904_RS06540 | hypothetical protein | -4.842 | 3.34E-05 |
| A5904_RS11175 | transketolase | -3.864 | 4.47E-05 |
| A5904_RS01720 | glucose-6-phosphate isomerase | -2.641 | 6.63E-05 |
| A5904_RS04150 | 1-phosphofructokinase family hexose kinase | -2.780 | 6.70E-05 |
| A5904_RS04160 | aldolase | -2.867 | 0.000832 |
| A5904_RS03905 | DNA/RNA nuclease SfsA | -1.944 | 0.001246 |
| A5904_RS11170 | hypothetical protein | -3.074 | 0.001489 |
| A5904_RS01185 | 1,4-alpha-glucan branching protein GlgB | -2.664 | 0.007458 |
| A5904_RS06995 | DMT family transporter | -1.669 | 0.011495 |
| A5904_RS11335 | transaldolase | -2.249 | 0.012510 |
| A5904_RS05205 | 2,3-bisphosphoglycerate-independent phosphoglycerate mutase | -1.855 | 0.015310 |
| A5904_RS05200 | phosphoenolpyruvate synthase | -2.364 | 0.017295 |
| novel0239 | transaldolase [Acidithiobacillus caldus] | -3.267 | 0.017650 |
| A5904_RS07145 | glucose-6-phosphate dehydrogenase | -2.276 | 0.019271 |
| A5904_RS07800 | inositol monophosphatase family protein | -1.527 | 0.020515 |
| A5904_RS07140 | decarboxylating 6-phosphogluconate dehydrogenase | -2.023 | 0.027916 |
| A5904_RS11695 | MFS transporter | -1.254 | 0.037602 |
| A5904_RS04165 | pyruvate kinase | -1.367 | 0.037618 |
| A5904_RS03640 | MFS transporter | -1.540 | 0.041879 |
| A5904_RS09150 | alpha-D-glucose phosphate-specific phosphoglucomutase | -1.836 | 0.048969 |
| **H: Coenzyme transport and metabolism** | | | |
| **UP** |  |  |  |
| A5904_RS04265 | pantoate--beta-alanine ligase | 4.175 | 5.81E-07 |
| A5904_RS10540 | phosphomethylpyrimidine synthase ThiC | 2.015 | 0.001829 |
| A5904_RS00845 | glutathione synthase | 2.016 | 0.002568 |
| A5904_RS04890 | 3-phosphoserine/phosphohydroxythreonine transaminase | 2.076 | 0.006749 |
| A5904_RS10335 | HesA/MoeB/ThiF family protein | 1.937 | 0.007091 |
| A5904_RS10785 | lipoate--protein ligase family protein | 1.704 | 0.008772 |
| A5904_RS09030 | 2-polyprenylphenol 6-hydroxylase | 1.709 | 0.010574 |
| A5904_RS10810 | lipoate--protein ligase family protein | 1.860 | 0.011495 |
| A5904_RS09845 | 1-deoxy-D-xylulose-5-phosphate synthase | 1.637 | 0.011521 |
| A5904_RS04260 | 3-methyl-2-oxobutanoate hydroxymethyltransferase | 2.366 | 0.013463 |
| A5904_RS13595 | glutamate-1-semialdehyde 2,1-aminomutase | 1.521 | 0.014337 |
| A5904_RS09960 | molybdenum cofactor guanylyltransferase | 1.552 | 0.015415 |
| A5904_RS11780 | dihydropteroate synthase | 1.884 | 0.017738 |
| A5904_RS09850 | polyprenyl synthetase family protein | 1.650 | 0.018774 |
| A5904_RS02225 | class I SAM-dependent methyltransferase | 1.764 | 0.020234 |
| A5904_RS09205 | class I SAM-dependent methyltransferase | 1.764 | 0.020234 |
| A5904_RS13600 | thiamine phosphate synthase | 1.472 | 0.047573 |
| **DOWN** |  |  |  |
| A5904_RS12265 | 8-amino-7-oxononanoate synthase | -2.368 | 0.001534 |
| A5904_RS03620 | class I SAM-dependent methyltransferase | -1.771 | 0.007765 |
| A5904_RS08650 | polyprenyl synthetase family protein | -1.813 | 0.007972 |
| A5904_RS12990 | oxygen-independent coproporphyrinogen III oxidase | -1.449 | 0.019954 |
| **I: Lipid transport and metabolism** | | | |
| **UP** |  |  |  |
| A5904_RS13915 | phospholipase D-like domain-containing protein | 2.188 | 0.015989 |
| **DOWN** |  |  |  |
| A5904_RS01895 | NAD(P)-dependent oxidoreductase | -2.767 | 0.004289 |
| A5904_RS04075 | phospholipase D-like domain-containing protein | -1.994 | 0.011694 |
| A5904_RS11685 | cyclopropane-fatty-acyl-phospholipid synthase family protein | -1.572 | 0.016681 |
| A5904_RS10220 | SCP2 sterol-binding domain-containing protein | -2.011 | 0.047300 |
| **J: Translation, ribosomal structure and biogenesis** | | | |
| **UP** |  |  |  |
| novel0185 | redox-regulated ATPase YchF  [Acidithiobacillus caldus] | 4.330 | 6.84E-09 |
| A5904_RS07745 | tRNA 2-thiouridine(34) synthase MnmA | 2.803 | 2.66E-05 |
| A5904_RS13010 | 30S ribosomal protein S12 methylthiotransferase RimO | 2.537 | 0.000439 |
| A5904_RS11420 | ribosome-binding factor A | 2.210 | 0.001576 |
| A5904_RS02880 | GTPase HflX | 2.192 | 0.002503 |
| A5904_RS11425 | translation initiation factor IF-2 | 1.700 | 0.002695 |
| A5904_RS11435 | ribosome maturation factor RimP | 1.714 | 0.005867 |
| A5904_RS11905 | phenylalanine--tRNA ligase subunit alpha | 1.533 | 0.012347 |
| A5904_RS08515 | L-threonylcarbamoyladenylate synthase | 2.027 | 0.016681 |
| A5904_RS06300 | L-threonylcarbamoyladenylate synthase | 2.328 | 0.032052 |
| A5904_RS01070 | lysine--tRNA ligase | 1.199 | 0.045470 |
| **DOWN** |  |  |  |
| novel0167 | MBL fold metallo-hydrolase  [Acidithiobacillus caldus] | -4.861 | 1.01E-06 |
| novel0191 | 50S ribosomal protein L10 [Acidithiobacillus caldus] | -3.100 | 0.001294 |
| A5904_RS11215 | sulfurtransferase TusA family protein | -2.483 | 0.001615 |
| A5904_RS10615 | translation elongation factor 4 | -2.069 | 0.003878 |
| A5904_RS12390 | division/cell wall cluster transcriptional repressor MraZ | -2.669 | 0.010012 |
| A5904_RS08465 | 50S ribosomal protein L25/general stress protein Ctc | -1.888 | 0.010081 |
| A5904_RS10870 | sulfurtransferase TusA family protein | -2.301 | 0.015581 |
| A5904_RS08935 | 30S ribosomal protein S10 | -2.261 | 0.015889 |
| A5904_RS08330 | valine--tRNA ligase | -1.371 | 0.018446 |
| A5904_RS07900 | 30S ribosomal protein S20 | -1.860 | 0.042830 |
| A5904_RS03950 | preQ(1) synthase | -1.286 | 0.044729 |
| **K: Transcription** | | | |
| **UP** |  |  |  |
| A5904_RS11655 | heat-inducible transcriptional repressor HrcA | 3.743 | 5.85E-05 |
| A5904_RS07300 | LysR family transcriptional regulator | 2.321 | 0.000555 |
| A5904_RS00600 | MarR family EPS-associated transcriptional regulator | 2.805 | 0.000811 |
| A5904_RS11430 | transcription termination factor NusA | 1.968 | 0.000811 |
| A5904_RS04180 | LysR family transcriptional regulator | 2.940 | 0.001059 |
| A5904_RS03730 | PadR family transcriptional regulator | 1.970 | 0.009305 |
| A5904_RS06900 | ribonuclease R | 1.244 | 0.020234 |
| A5904_RS03220 | transcriptional repressor LexA | 2.172 | 0.022489 |
| A5904_RS11530 | heavy metal-responsive transcriptional regulator | 2.234 | 0.032933 |
| A5904_RS02775 | MarR family transcriptional regulator | 1.398 | 0.040911 |
| A5904_RS09195 | metalloregulator ArsR/SmtB family transcription factor | 1.849 | 0.043638 |
| A5904_RS02235 | metalloregulator ArsR/SmtB family transcription factor | 1.849 | 0.043638 |
| A5904_RS11725 | MerR family transcriptional regulator | 1.396 | 0.044840 |
| A5904_RS14190 | TraR/DksA C4-type zinc finger protein | 1.460 | 0.045681 |
| **DOWN** |  |  |  |
| A5904_RS02335 | sigma-70 family RNA polymerase sigma factor | -7.469 | 6.19E-26 |
| A5904_RS03720 | RNA polymerase-binding protein DksA | -2.830 | 6.54E-05 |
| novel0260 | TetR family transcriptional regulator  [Acidithiobacillus caldus] | -3.626 | 0.000124 |
| A5904_RS01645 | AbrB/MazE/SpoVT family DNA-binding domain-containing protein | -2.686 | 0.005045 |
| A5904_RS08795 | DNA-directed RNA polymerase subunit alpha | -2.237 | 0.007458 |
|  |  |  |  |
| novel0219 | RNA polymerase sigma factor RpoE  [Acidithiobacillus caldus] | -2.702 | 0.044761 |
| **L: Replication, recombination and repair** | | | |
| **UP** |  |  |  |
| novel0250 | Smr/MutS family protein [Acidithiobacillus caldus] | 3.562 | 6.68E-08 |
| A5904_RS02445 | DEAD/DEAH box helicase | 2.757 | 3.48E-06 |
| A5904_RS10430 | Smr/MutS family protein | 3.334 | 1.34E-05 |
| A5904_RS04275 | Smr/MutS family protein | 3.382 | 4.08E-05 |
| A5904_RS06950 | exonuclease domain-containing protein | 2.792 | 7.66E-05 |
| A5904_RS01395 | endonuclease III | 2.138 | 0.000428 |
| A5904_RS09675 | site-specific integrase | 3.284 | 0.000724 |
| A5904_RS00005 | chromosomal replication initiator protein DnaA | 2.392 | 0.001236 |
| A5904_RS00010 | DNA polymerase III subunit beta | 2.201 | 0.002032 |
| A5904_RS00660 | DNA repair protein RadA | 2.258 | 0.002984 |
| A5904_RS04850 | integration host factor subunit beta | 2.499 | 0.005853 |
| A5904_RS00020 | DNA topoisomerase (ATP-hydrolyzing) subunit B | 1.621 | 0.009824 |
| A5904_RS02505 | integrase family protein | 2.142 | 0.009988 |
| A5904_RS04895 | DNA gyrase subunit A | 2.080 | 0.013984 |
| A5904_RS00050 | type I DNA topoisomerase | 1.487 | 0.015813 |
| A5904_RS09105 | methylated-DNA--[protein]-cysteine S-methyltransferase | 1.629 | 0.016681 |
| A5904_RS13955 | ATP-dependent helicase | 1.396 | 0.018887 |
| A5904_RS00015 | DNA replication and repair protein RecF | 2.102 | 0.020030 |
| A5904_RS14270 | tyrosine-type recombinase/integrase | 1.507 | 0.029028 |
| A5904_RS00780 | exodeoxyribonuclease V subunit gamma | 1.664 | 0.042830 |
| **DOWN** |  |  |  |
| novel0035 | primosomal protein N', partial [Acidithiobacillus caldus] | -5.082 | 2.07E-09 |
| novel0105 | DNA gyrase subunit A, partial [Acidithiobacillus caldus] | -3.087 | 0.000513 |
| A5904_RS09365 | DNA-3-methyladenine glycosylase | -2.228 | 0.001193 |
| A5904_RS05750 | AAA family ATPase | -2.015 | 0.007091 |
| A5904_RS15175 | HU family DNA-binding protein | -2.121 | 0.012862 |
| A5904_RS04285 | DUF3820 family protein | -1.537 | 0.019271 |
| A5904_RS09285 | DNA polymerase IV | -1.597 | 0.041191 |
| A5904_RS06255 | site-specific integrase | -1.237 | 0.047969 |
| **M: Cell wall/membrane/envelope biogenesis** | | | |
| **UP** |  |  |  |
| A5904_RS10460 | DUF481 domain-containing protein | 3.867 | 5.27E-08 |
| A5904_RS04685 | PBP1A family penicillin-binding protein | 3.320 | 2.73E-06 |
| A5904_RS00575 | GDP-mannose 4,6-dehydratase | 3.543 | 2.59E-05 |
| A5904_RS03190 | HAD-IIB family hydrolase | 2.576 | 0.000110 |
| A5904_RS01865 | mechanosensitive ion channel | 2.568 | 0.000438 |
| A5904_RS13315 | carbohydrate porin | 2.609 | 0.001214 |
| A5904_RS10495 | glucan biosynthesis protein | 2.955 | 0.001236 |
| A5904_RS00585 | WbeA | 2.906 | 0.001294 |
| A5904_RS12365 | UDP-3-O-acyl-N-acetylglucosamine deacetylase | 3.033 | 0.002908 |
| A5904_RS10485 | glucans biosynthesis glucosyltransferase MdoH | 2.281 | 0.004339 |
| A5904_RS00580 | NAD-dependent epimerase/dehydratase family protein | 2.611 | 0.005634 |
| A5904_RS03180 | mechanosensitive ion channel | 2.426 | 0.007698 |
| A5904_RS00665 | alanine racemase | 1.823 | 0.031445 |
| A5904_RS11325 | LysM peptidoglycan-binding domain-containing protein | 1.288 | 0.036364 |
| A5904_RS01580 | LPS assembly lipoprotein LptE | 1.565 | 0.040372 |
| A5904_RS11350 | UDP- 2,3-diacylglucosamine diphosphatase | 1.595 | 0.042402 |
| A5904_RS10245 | HpnM family protein | 1.359 | 0.047114 |
| **DOWN** |  |  |  |
| novel0202 | efflux transporter outer membrane subunit [Acidithiobacillus caldus] | -4.342 | 5.69E-06 |
| A5904_RS03215 | host attachment protein | -3.874 | 1.85E-05 |
| A5904_RS14315 | Slp family lipoprotein | -2.257 | 0.000110 |
| A5904_RS15365 | SLBB domain-containing protein | -2.273 | 0.000964 |
| A5904_RS01785 | lipoprotein insertase outer membrane protein LolB | -2.549 | 0.001085 |
| A5904_RS10965 | rod shape-determining protein MreD | -2.096 | 0.001399 |
| A5904_RS12585 | outer membrane protein assembly factor BamD | -2.650 | 0.005045 |
| A5904_RS15485 | hypothetical protein | -1.956 | 0.007562 |
| A5904_RS12975 | OmpA family protein | -1.779 | 0.014889 |
| A5904_RS00980 | substrate-binding domain-containing protein | -2.778 | 0.018114 |
| A5904_RS07940 | efflux RND transporter periplasmic adaptor subunit | -2.602 | 0.022890 |
| A5904_RS08415 | MlaD family protein | -1.396 | 0.023703 |
| A5904_RS08555 | Slp family lipoprotein | -2.547 | 0.029733 |
| A5904_RS05120 | NAD-dependent epimerase/dehydratase family protein | -1.282 | 0.031757 |
| **N: Cell motility** | | | |
| **DOWN** |  |  |  |
| A5904_RS04430 | flagellin | -3.607 | 1.25E-09 |
| novel0095 | flagellar protein FlaB [Acidithiobacillus caldus] | -4.052 | 1.27E-06 |
| A5904_RS04530 | flagellar motor protein MotD | -3.122 | 2.19E-06 |
| A5904_RS04745 | prepilin-type N-terminal cleavage/methylation domain-containing protein | -3.325 | 0.000196 |
| A5904_RS06790 | PilZ domain-containing protein | -2.490 | 0.001829 |
| A5904_RS04495 | flagellar basal body rod protein FlgB | -3.859 | 0.003863 |
| A5904_RS04755 | prepilin-type N-terminal cleavage/methylation domain-containing protein | -2.500 | 0.013778 |
| A5904_RS04040 | flagellar motor protein MotB | -2.425 | 0.019576 |
| A5904_RS01940 | hypothetical protein | -1.820 | 0.021225 |
| A5904_RS12150 | type IV pilus twitching motility protein PilT | -1.422 | 0.023196 |
| A5904_RS04440 | flagellar hook-associated protein FlgL | -2.089 | 0.029154 |
| A5904_RS04480 | flagellar hook protein FlgE | -2.190 | 0.031970 |
| A5904_RS04700 | hypothetical protein | -1.090 | 0.040483 |
| A5904_RS12145 | PilT/PilU family type 4a pilus ATPase | -1.168 | 0.041135 |
| A5904_RS04415 | flagellar export chaperone FliS | -2.136 | 0.044274 |
| **O: Posttranslational modification, protein turnover, chaperones** | | | |
| **UP** |  |  |  |
| A5904_RS07760 | Fe-S protein assembly chaperone HscA | 3.248 | 8.01E-05 |
| A5904_RS07765 | Fe-S protein assembly co-chaperone HscB | 3.122 | 0.000855 |
| A5904_RS02240 | glutathione S-transferase | 2.552 | 0.001036 |
| A5904_RS09190 | glutathione S-transferase | 2.552 | 0.001036 |
| A5904_RS10115 | chaperonin GroEL | 2.539 | 0.001872 |
| A5904_RS03915 | [protein-PII] uridylyltransferase | 2.136 | 0.002595 |
| A5904_RS11560 | glutathione peroxidase | 2.227 | 0.009473 |
| A5904_RS10110 | co-chaperone GroES | 2.079 | 0.022883 |
| A5904_RS02215 | TlpA disulfide reductase family protein | 2.544 | 0.027009 |
| A5904_RS12285 | 5-histidylcysteine sulfoxide synthase | 1.971 | 0.028778 |
| A5904_RS12240 | leucyl/phenylalanyl-tRNA--protein transferase | 1.541 | 0.042803 |
| **DOWN** |  |  |  |
| A5904_RS08160 | Fe-S cluster assembly scaffold IscU | -3.619 | 3.03E-08 |
| A5904_RS10705 | thiol:disulfide interchange protein DsbG | -3.537 | 2.59E-07 |
| A5904_RS09350 | thioredoxin family protein | -3.358 | 3.52E-07 |
| A5904_RS12095 | thioredoxin fold domain-containing protein | -3.463 | 2.12E-06 |
| A5904_RS15745 | hypothetical protein | -2.921 | 4.56E-06 |
| A5904_RS01765 | PepSY domain-containing protein | -3.139 | 0.000750 |
| A5904_RS13330 | Hsp20/alpha crystallin family protein | -3.096 | 0.001052 |
| novel0156 | TlpA family protein disulfide reductase [Acidithiobacillus caldus] | -2.828 | 0.003896 |
| A5904_RS13530 | ClpXP protease specificity-enhancing factor | -2.062 | 0.006632 |
| A5904_RS01180 | hypothetical protein | -1.980 | 0.010302 |
| A5904_RS01175 | SPFH/Band 7/PHB domain protein | -2.147 | 0.011516 |
| A5904_RS07190 | TlpA disulfide reductase family protein | -2.229 | 0.014673 |
| novel0285 | Hsp20/alpha crystallin family protein [Acidithiobacillus caldus] | -2.795 | 0.019881 |
| A5904_RS00260 | DnaJ domain-containing protein | -1.737 | 0.032607 |
| A5904_RS09255 | thioredoxin fold domain-containing protein | -1.881 | 0.044815 |
| A5904_RS10465 | molecular chaperone HtpG | -1.436 | 0.045709 |
| **P: Inorganic ion transport and metabolism** | | | |
| **UP** |  |  |  |
| A5904_RS00870 | ferrous iron transport protein B | 4.056 | 1.27E-06 |
| A5904_RS07065 | sulfite exporter TauE/SafE family protein | 2.914 | 0.000327 |
| A5904_RS04925 | phosphate ABC transporter substrate-binding protein PstS | 1.650 | 0.006800 |
| A5904_RS00645 | hypothetical protein | 2.530 | 0.016183 |
| A5904_RS10760 | VWA domain-containing protein | 1.279 | 0.029864 |
| novel0256 | ferric iron uptake transcriptional regulator [Acidithiobacillus caldus] | 2.135 | 0.039888 |
| A5904_RS07230 | VWA domain-containing protein | 1.974 | 0.042009 |
| **DOWN** |  |  |  |
| A5904_RS06455 | extracellular solute-binding protein | -4.301 | 2.00E-10 |
| novel0284 | cation ABC transporter substrate-binding protein, partial [Acidithiobacillus caldus] | -4.352 | 4.64E-08 |
| A5904_RS03165 | phosphate ABC transporter substrate-binding protein PstS | -3.011 | 8.65E-06 |
| A5904_RS00940 | extracellular solute-binding protein | -4.010 | 2.59E-05 |
| A5904_RS04655 | ABC transporter permease subunit | -2.867 | 3.67E-05 |
| A5904_RS03125 | DsrE family protein | -3.040 | 0.000191 |
| A5904_RS08260 | hypothetical protein | -2.862 | 0.000978 |
| A5904_RS02410 | ammonium transporter | -1.653 | 0.003901 |
| A5904_RS06700 | mercuric transporter MerT family protein | -1.846 | 0.008963 |
| A5904_RS00925 | potassium-transporting ATPase subunit KdpA | -1.596 | 0.010630 |
| A5904_RS06705 | heavy metal-associated domain-containing protein | -1.949 | 0.011951 |
| A5904_RS01505 | oxidative damage protection protein | -1.595 | 0.015023 |
| A5904_RS06415 | NAD-binding protein | -1.759 | 0.040372 |
| **Q: Secondary metabolites biosynthesis** | | | |
| **UP** |  |  |  |
| A5904_RS07270 | carboxysome peptide B | 3.481 | 0.000397 |
| **R: General function prediction only** | | | |
| **UP** |  |  |  |
| A5904_RS14660 | ATP-binding protein | 2.832 | 4.68E-05 |
| A5904_RS03725 | DUF2202 domain-containing protein | 2.125 | 0.001085 |
| A5904_RS10435 | GatB/YqeY domain-containing protein | 2.848 | 0.001147 |
| A5904_RS10265 | MMPL family transporter | 2.021 | 0.001680 |
| A5904_RS09540 | AI-2E family transporter | 2.134 | 0.003055 |
| A5904_RS10815 | radical SAM protein | 1.846 | 0.003593 |
| A5904_RS10275 | hopanoid biosynthesis associated radical SAM protein HpnJ | 1.985 | 0.012225 |
| A5904_RS08285 | EVE domain-containing protein | 1.440 | 0.024621 |
| A5904_RS11770 | metalloprotease TldD | 2.164 | 0.025005 |
| A5904_RS11400 | pitrilysin family protein | 1.455 | 0.026273 |
| **DOWN** |  |  |  |
| A5904_RS09355 | hemerythrin domain-containing protein | -4.485 | 3.54E-10 |
| novel0110 | alpha/beta hydrolase, partial  [Acidithiobacillus caldus] | -4.061 | 7.26E-09 |
| A5904_RS11755 | YbhB/YbcL family Raf kinase inhibitor-like protein | -3.462 | 3.44E-05 |
| A5904_RS05210 | carboxymuconolactone decarboxylase family protein | -2.480 | 0.000499 |
| A5904_RS09555 | AI-2E family transporter | -2.858 | 0.000730 |
| A5904_RS15590 | OsmC family protein | -2.611 | 0.003222 |
| novel0213 | MMPL family transporter, partial  [Acidithiobacillus caldus] | -3.138 | 0.003979 |
| A5904_RS07000 | hypothetical protein | -1.700 | 0.005334 |
| A5904_RS09395 | hemerythrin domain-containing protein | -2.006 | 0.009182 |
| A5904_RS12910 | pilus assembly protein TadG-related protein | -1.712 | 0.010630 |
| A5904_RS04580 | hypothetical protein | -2.709 | 0.011694 |
| A5904_RS13135 | FmdB family transcriptional regulator | -2.147 | 0.013643 |
| **S: Function unknown** | | | |
| **UP** |  |  |  |
| A5904_RS02830 | hypothetical protein | 2.861 | 6.38E-05 |
| A5904_RS02840 | hypothetical protein | 3.152 | 0.002155 |
| A5904_RS07425 | DUF302 domain-containing protein | 2.355 | 0.002211 |
| A5904_RS15585 | SHOCT domain-containing protein | 2.630 | 0.003170 |
| A5904_RS15415 | SHOCT domain-containing protein | 2.630 | 0.003170 |
| A5904_RS10555 | phosphate-starvation-inducible PsiE family protein | 1.869 | 0.012188 |
| A5904_RS15300 | DUF2384 domain-containing protein | 1.440 | 0.014945 |
| A5904_RS06945 | DUF2237 domain-containing protein | 2.442 | 0.015030 |
| A5904_RS00790 | DUF692 domain-containing protein | 1.788 | 0.028520 |
| A5904_RS07315 | DUF202 domain-containing protein | 1.901 | 0.031244 |
| **DOWN** |  |  |  |
| A5904_RS09430 | DUF488 family protein | -3.585 | 4.14E-07 |
| A5904_RS11225 | hypothetical protein | -2.882 | 0.000387 |
| A5904_RS05280 | YbjQ family protein | -2.005 | 0.002114 |
| A5904_RS07085 | ferritin-like domain-containing protein | -2.166 | 0.004330 |
| A5904_RS00975 | hypothetical protein | -3.059 | 0.005207 |
| A5904_RS08390 | AbrB/MazE/SpoVT family DNA-binding domain-containing protein | -1.877 | 0.019871 |
| A5904_RS06615 | DUF2282 domain-containing protein | -2.191 | 0.020777 |
|  | **T: Signal transduction mechanisms** |  |  |
| **UP** |  |  |  |
| A5904_RS02640 | tryptophan-rich sensory protein | 2.024 | 0.002898 |
| A5904_RS00650 | EAL domain-containing protein | 1.772 | 0.002984 |
| A5904_RS00085 | ATP-binding protein | 2.247 | 0.003863 |
| A5904_RS00090 | sigma-54 dependent transcriptional regulator | 2.464 | 0.004142 |
| A5904_RS09670 | DUF190 domain-containing protein | 2.049 | 0.009305 |
| **DOWN** |  |  |  |
| A5904_RS10070 | universal stress protein | -4.076 | 1.27E-08 |
| A5904_RS08240 | sigma-54 dependent transcriptional regulator | -3.227 | 4.14E-07 |
| novel0269 | homoserine kinase, partial [Acidithiobacillus caldus] | -4.765 | 5.46E-07 |
| A5904_RS04540 | chemotaxis response regulator CheY | -4.365 | 2.18E-05 |
| novel0221 | FIST C-terminal domain-containing protein  [Acidithiobacillus caldus] | -3.271 | 0.000470 |
| A5904_RS05320 | CBS domain-containing protein | -2.439 | 0.000664 |
| A5904_RS15645 | HipA N-terminal domain-containing protein | -3.222 | 0.001230 |
| A5904_RS04065 | GGDEF domain-containing protein | -1.747 | 0.003437 |
| A5904_RS09890 | CBS domain-containing protein | -2.153 | 0.003959 |
| A5904_RS03245 | response regulator | -1.471 | 0.008963 |
| A5904_RS00280 | response regulator | -1.908 | 0.009589 |
| A5904_RS01760 | response regulator | -1.904 | 0.017075 |
| A5904_RS03240 | ATP-binding protein | -1.452 | 0.022639 |
| A5904_RS04035 | EAL domain-containing protein | -1.242 | 0.045435 |
| novel0022 | two-component sensor histidine kinase  [Acidithiobacillus caldus] | -2.147 | 0.045709 |
| **U: Intracellular trafficking, secretion, and vesicular transport** | | | |
| **UP** |  |  |  |
| A5904_RS09865 | protein translocase subunit SecD | 1.296 | 0.017786 |
| A5904_RS09135 | signal recognition particle protein | 2.180 | 0.020670 |
| **DOWN** |  |  |  |
| A5904_RS13190 | protein TolR | -2.234 | 0.004467 |
| A5904_RS10610 | signal peptidase I | -1.875 | 0.006800 |
| A5904_RS01230 | Sec-independent protein translocase subunit TatA | -2.891 | 0.007792 |
| A5904_RS01095 | MotA/TolQ/ExbB proton channel family protein | -1.528 | 0.015958 |
| novel0188 | MotA/TolQ/ExbB proton channel family protein [Acidithiobacillus caldus] | -2.578 | 0.020186 |
| novel0016 | Sec-independent protein translocase subunit TatA [Acidithiobacillus caldus] | -2.747 | 0.021733 |
| **V: Defense mechanisms** | | | |
| **UP** |  |  |  |
| A5904_RS11470 | ABC transporter ATP-binding protein | 3.761 | 2.01E-06 |
| A5904_RS11110 | peroxiredoxin | 2.653 | 7.40E-05 |
| A5904_RS01655 | type II toxin-antitoxin system VapC family toxin | 1.947 | 0.010575 |
| A5904_RS06490 | hypothetical protein | 2.098 | 0.019397 |
| A5904_RS10405 | Txe/YoeB family addiction module toxin | 2.472 | 0.021446 |
| A5904_RS12185 | HlyD family secretion protein | 1.376 | 0.022489 |
| A5904_RS07200 | NUDIX hydrolase | 1.447 | 0.027581 |
| A5904_RS14150 | RRXRR domain-containing protein | 1.443 | 0.037940 |
| **DOWN** |  |  |  |
| novel0205 | ABC transporter permease [Acidithiobacillus caldus] | -5.692 | 0.001333 |
| A5904_RS12490 | DJ-1/PfpI family protein | -2.820 | 0.001829 |
| novel0106 | efflux RND transporter periplasmic adaptor subunit, partial [Acidithiobacillus caldus] | -3.143 | 0.013909 |
| A5904_RS13400 | HNH endonuclease | -1.692 | 0.029165 |
| **X: Mobilome: prophages, transposons** | | | |
| **UP** |  |  |  |
| novel0290 | MULTISPECIES: ISL3 family transposase  [Acidithiobacillus] | 2.925 | 6.39E-07 |
| novel0307 | transposase [Acidithiobacillus caldus] | 3.513 | 8.40E-05 |
| A5904_RS00570 | IS5 family transposase | 2.578 | 0.000163 |
| A5904_RS03550 | IS66 family transposase | 2.236 | 0.000970 |
| A5904_RS13090 | IS66 family transposase | 2.340 | 0.002974 |
| A5904_RS04630 | IS66 family transposase | 2.340 | 0.002974 |
| A5904_RS09310 | IS66 family transposase | 2.340 | 0.002974 |
| A5904_RS02810 | IS66 family transposase | 2.340 | 0.002974 |
| A5904_RS06740 | IS66 family transposase | 2.340 | 0.002974 |
| A5904_RS03260 | IS66 family transposase | 2.340 | 0.002974 |
| A5904_RS07405 | IS5 family transposase | 1.509 | 0.008315 |
| A5904_RS02555 | IS630 family transposase | 2.572 | 0.010324 |
| A5904_RS00475 | type II toxin-antitoxin system RelE/ParE family toxin | 2.175 | 0.015834 |
| A5904_RS13895 | type II toxin-antitoxin system mRNA interferase toxin, RelE/StbE family | 1.725 | 0.019170 |
| A5904_RS08135 | IS5 family transposase | 1.580 | 0.020803 |
| novel0337 | transposase, partial [Acidithiobacillus thiooxidans] | 7.618 | 0.023703 |
| A5904_RS03580 | IS66 family transposase | 1.761 | 0.025599 |
| A5904_RS03475 | IS66 family transposase | 1.761 | 0.025599 |
| novel0291 | transposase, partial [Acidithiobacillus caldus] | 2.292 | 0.029048 |
| novel0353 | transposase, partial [Acidithiobacillus thiooxidans] | 10.307 | 0.042803 |
| novel0339 | ISAtc1-like protein [Acidithiobacillus caldus] | 10.129 | 0.046931 |
| **DOWN** |  |  |  |
| novel0079 | IS66 family transposase [Acidithiobacillus caldus] | -3.121 | 3.52E-07 |
| novel0341 | ISL3 family transposase, partial [Acidithiobacillus caldus] | -3.085 | 2.08E-06 |
| novel0211 | IS21 family transposase [Acidithiobacillus caldus] | -3.215 | 4.20E-06 |
| novel0172 | MULTISPECIES: ISL3 family transposase [Acidithiobacillus] | -2.761 | 9.95E-05 |
| novel0127 | MULTISPECIES: ISL3 family transposase [Acidithiobacillus] | -3.387 | 0.000368 |
| novel0053 | transposase [Acidithiobacillus caldus] | -4.401 | 0.002155 |
| novel0318 | transposase [Acidithiobacillus caldus] | -4.401 | 0.002155 |
| A5904_RS01945 | IS1595 family transposase | -2.648 | 0.004995 |
| novel0208 | MULTISPECIES: ISL3 family transposase [Acidithiobacillus] | -12.987 | 0.007765 |
| A5904_RS08485 | ISL3-like element ISAtc1 family transposase | -1.819 | 0.012347 |
| novel0201 | ISL3-like element ISAtc2 family transposase [Acidithiobacillus caldus] | -10.429 | 0.020234 |
| A5904_RS06190 | transposase | -3.332 | 0.043964 |
| novel0138 | IS66 family transposase [Acidithiobacillus caldus] | -1.674 | 0.046903 |
| **Other** | | | |
| **UP** |  |  |  |
| A5904_RS15430 | DUF4168 domain-containing protein | 6.722 | 1.57E-24 |
| A5904_RS02815 | hypothetical protein | 13.685 | 3.28E-19 |
| novel0046 | conserved hypothetical protein [Acidithiobacillus caldus SM-1] | 5.366 | 6.80E-11 |
| A5904_RS06690 | hypothetical protein | 9.761 | 1.30E-09 |
| A5904_RS00875 | FeoA domain-containing protein | 4.837 | 3.77E-07 |
| A5904_RS09680 | DUF4160 domain-containing protein | 4.193 | 4.11E-07 |
| novel0342 | hypothetical protein [Acidithiobacillus caldus] | 5.355 | 4.31E-06 |
| novel0177 | hypothetical protein [Acidithiobacillus caldus] | 5.355 | 4.31E-06 |
| A5904_RS09690 | hypothetical protein | 3.933 | 1.00E-05 |
| A5904_RS13960 | hypothetical protein | 3.125 | 1.16E-05 |
| A5904_RS02220 | MarR family transcriptional regulator | 3.105 | 2.66E-05 |
| novel0261 | DciA family protein, partial [Acidithiobacillus caldus] | 4.054 | 2.79E-05 |
| A5904_RS15030 | DNA-binding protein | 3.668 | 4.98E-05 |
| A5904_RS15445 | DUF4168 domain-containing protein | 3.509 | 0.000188 |
| A5904_RS14125 | hypothetical protein | 2.758 | 0.000767 |
| novel0210 | MULTISPECIES: hypothetical protein [unclassified Acidithiobacillus] | 3.083 | 0.001351 |
| A5904_RS00850 | glutamate--cysteine ligase | 2.394 | 0.001379 |
| A5904_RS02510 | hypothetical protein | 2.689 | 0.001615 |
| A5904_RS14255 | zinc ribbon domain-containing protein | 2.725 | 0.002155 |
| A5904_RS14260 | ProQ/FINO family protein | 2.145 | 0.002974 |
| A5904_RS07280 | carboxysome shell carbonic anhydrase | 2.274 | 0.002984 |
| A5904_RS01150 | flagellar motor protein MotB | 2.777 | 0.003165 |
| A5904_RS00865 | hypothetical protein | 2.588 | 0.003165 |
| A5904_RS10490 | hypothetical protein | 2.639 | 0.003700 |
| A5904_RS06235 | GNAT family N-acetyltransferase | 2.602 | 0.004467 |
| A5904_RS15010 | AbrB family transcriptional regulator | 2.283 | 0.006461 |
| A5904_RS15205 | hypothetical protein | 2.597 | 0.007091 |
| A5904_RS10340 | hypothetical protein | 2.191 | 0.007433 |
| A5904_RS09110 | hypothetical protein | 1.687 | 0.008011 |
| A5904_RS09210 | MarR family transcriptional regulator | 6.743 | 0.009490 |
| A5904_RS15640 | prepilin peptidase | 3.100 | 0.010575 |
| A5904_RS05425 | hypothetical protein | 2.072 | 0.011850 |
| A5904_RS11295 | hypothetical protein | 2.085 | 0.011968 |
| A5904_RS10330 | hypothetical protein | 2.297 | 0.012225 |
| novel0262 | hypothetical protein, partial  [Acidithiobacillus caldus] | 4.456 | 0.012694 |
| A5904_RS06910 | AAA family ATPase | 1.588 | 0.015023 |
| novel0176 | EexN family lipoprotein [Acidithiobacillus caldus] | 2.961 | 0.015415 |
| novel0346 | EexN family lipoprotein [Acidithiobacillus caldus] | 2.961 | 0.015415 |
| A5904_RS06450 | YrhK family protein | 2.253 | 0.016329 |
| A5904_RS05935 | hypothetical protein | 2.142 | 0.018686 |
| A5904_RS15785 | hypothetical protein | 2.548 | 0.021733 |
| A5904_RS10790 | hypothetical protein | 2.382 | 0.022489 |
| A5904_RS03145 | hypothetical protein | 1.977 | 0.022984 |
| A5904_RS07575 | hypothetical protein | 2.308 | 0.025229 |
| A5904_RS02985 | hypothetical protein | 1.765 | 0.025654 |
| A5904_RS06090 | hypothetical protein | 1.578 | 0.028520 |
| A5904_RS14060 | hypothetical protein | 2.361 | 0.029165 |
| A5904_RS15790 | hypothetical protein | 2.541 | 0.029864 |
| A5904_RS09145 | hypothetical protein | 1.621 | 0.030064 |
| novel0062 | DUF4168 domain-containing protein [Acidithiobacillus caldus] | 3.448 | 0.030860 |
| A5904_RS07195 | BMC domain-containing protein | 1.663 | 0.040483 |
| A5904_RS06925 | phosphate-starvation-inducible PsiE family protein | 1.392 | 0.040483 |
| A5904_RS01215 | hypothetical protein | 2.166 | 0.042803 |
| A5904_RS02550 | hypothetical protein | 1.465 | 0.043783 |
| A5904_RS09140 | diiron oxygenase | 1.456 | 0.044710 |
| A5904_RS14065 | ribbon-helix-helix protein, CopG family | 1.737 | 0.045709 |
| **DOWN** |  |  |  |
| A5904_RS02340 | hypothetical protein | -6.586 | 5.26E-20 |
| A5904_RS02345 | hypothetical protein | -5.980 | 2.30E-17 |
| A5904_RS07930 | hypothetical protein | -4.810 | 2.54E-13 |
| A5904_RS02350 | hypothetical protein | -5.474 | 5.18E-13 |
| A5904_RS02115 | hypothetical protein | -4.078 | 1.73E-10 |
| A5904_RS09165 | hypothetical protein | -4.758 | 3.54E-10 |
| A5904_RS00305 | hypothetical protein | -4.269 | 3.67E-10 |
| A5904_RS14275 | glycine zipper domain-containing protein | -4.207 | 1.00E-09 |
| A5904_RS02360 | hypothetical protein | -4.853 | 1.36E-09 |
| A5904_RS02355 | hypothetical protein | -4.650 | 4.85E-09 |
| A5904_RS15425 | hypothetical protein | -4.040 | 2.10E-08 |
| A5904_RS07580 | hypothetical protein | -5.080 | 5.78E-08 |
| A5904_RS13575 | hypothetical protein | -4.614 | 1.04E-07 |
| A5904_RS07935 | hypothetical protein | -3.694 | 1.39E-07 |
| novel0044 | sigma-70 family RNA polymerase sigma factor [Acidithiobacillus caldus] | -7.734 | 2.00E-07 |
| A5904_RS03390 | hypothetical protein | -4.312 | 6.36E-07 |
| A5904_RS07925 | hypothetical protein | -4.487 | 8.25E-07 |
| A5904_RS15105 | cytochrome P460 family protein | -3.084 | 9.47E-07 |
| novel0232 | hypothetical protein, partial [Acidithiobacillus caldus] | -3.790 | 2.91E-06 |
| novel0004 | two-component sensor histidine kinase [Acidithiobacillus caldus] | -4.300 | 3.95E-06 |
| A5904_RS07560 | DUF3501 family protein | -3.904 | 5.11E-06 |
| A5904_RS00670 | hypothetical protein | -3.079 | 6.45E-06 |
| A5904_RS04575 | hypothetical protein | -4.334 | 1.18E-05 |
| novel0015 | 1,4-alpha-glucan (glycogen) branching enzyme, GH-13-type [Acidithiobacillus caldus SM-1] | -4.861 | 1.70E-05 |
| A5904_RS12920 | hypothetical protein | -3.337 | 2.16E-05 |
| A5904_RS02495 | hypothetical protein | -2.878 | 2.26E-05 |
| A5904_RS10010 | hypothetical protein | -3.218 | 2.71E-05 |
| A5904_RS08325 | hypothetical protein | -2.801 | 3.50E-05 |
| A5904_RS04100 | hypothetical protein | -3.146 | 6.43E-05 |
| A5904_RS05300 | hypothetical protein | -3.660 | 0.000121 |
| A5904_RS01705 | hypothetical protein | -3.273 | 0.000199 |
| novel0347 | SLBB domain-containing protein [Acidithiobacillus caldus] | -3.604 | 0.000207 |
| A5904_RS02660 | hypothetical protein | -3.432 | 0.000210 |
| A5904_RS00285 | Spy/CpxP family protein refolding chaperone | -3.117 | 0.000217 |
| novel0238 | hypothetical protein BAE30_03715, partial [Acidithiobacillus caldus] | -5.004 | 0.000269 |
| novel0349 | hypothetical protein Acaty_m0137 [Acidithiobacillus caldus ATCC 51756] | -3.699 | 0.000306 |
| novel0114 | hypothetical protein [Acidithiobacillus caldus] | -3.910 | 0.000336 |
| A5904_RS07955 | hypothetical protein | -3.066 | 0.000368 |
| A5904_RS03625 | hypothetical protein | -3.471 | 0.000387 |
| A5904_RS15795 | hypothetical protein | -3.594 | 0.000587 |
| A5904_RS04410 | PilZ domain-containing protein | -2.609 | 0.001069 |
| novel0108 | hypothetical protein [Acidithiobacillus caldus] | -3.808 | 0.001238 |
| A5904_RS05690 | hypothetical protein | -3.374 | 0.001470 |
| A5904_RS11700 | DUF3833 domain-containing protein | -2.095 | 0.001615 |
| novel0020 | hypothetical protein, partial [Acidithiobacillus caldus] | -4.079 | 0.001793 |
| A5904_RS06080 | hypothetical protein | -3.048 | 0.001854 |
| A5904_RS15275 | hypothetical protein | -3.131 | 0.001871 |
| A5904_RS07585 | tetratricopeptide repeat protein | -2.980 | 0.001992 |
| A5904_RS11960 | hypothetical protein | -3.032 | 0.002289 |
| novel0152 | glucose-6-phosphate dehydrogenase [Acidithiobacillus caldus] | -3.780 | 0.002500 |
| A5904_RS04450 | hypothetical protein | -3.289 | 0.003437 |
| A5904_RS07965 | tetratricopeptide repeat protein | -2.572 | 0.003689 |
| A5904_RS01685 | hypothetical protein | -2.295 | 0.003979 |
| novel0092 | glutamate decarboxylase, partial [Acidithiobacillus caldus] | -3.533 | 0.004507 |
| A5904_RS10530 | hypothetical protein | -2.520 | 0.004887 |
| novel0087 | FdhF/YdeP family oxidoreductase [Acidithiobacillus caldus] | -3.520 | 0.005334 |
| novel0189 | outer membrane beta-barrel protein [Acidithiobacillus caldus] | -3.797 | 0.006321 |
| A5904_RS09260 | hypothetical protein | -2.649 | 0.006651 |
| A5904_RS01680 | hypothetical protein | -3.225 | 0.007221 |
| A5904_RS14695 | hypothetical protein | -2.925 | 0.007326 |
| novel0021 | hypothetical protein [Acidithiobacillus caldus] | -3.228 | 0.008348 |
| A5904_RS09535 | hypothetical protein | -2.124 | 0.009259 |
| A5904_RS15015 | hypothetical protein | -2.618 | 0.009259 |
| A5904_RS09085 | hypothetical protein | -1.854 | 0.009259 |
| novel0319 | conserved hypothetical protein [Acidithiobacillus caldus SM-1] | -3.575 | 0.009473 |
| A5904_RS08150 | hypothetical protein | -2.329 | 0.010121 |
| novel0268 | DJ-1/PfpI family protein, partial [Acidithiobacillus caldus] | -4.871 | 0.010302 |
| A5904_RS04505 | flagellar biosynthesis anti-sigma factor FlgM | -2.173 | 0.010343 |
| novel0043 | hypothetical protein BAE27_11265 [Acidithiobacillus caldus] | -3.242 | 0.010787 |
| A5904_RS07970 | hypothetical protein | -1.979 | 0.011495 |
| novel0066 | DsrE family protein [Acidithiobacillus caldus] | -4.876 | 0.011495 |
| novel0320 | transposase [Acidithiobacillus caldus] | -2.905 | 0.013611 |
| A5904_RS03525 | hypothetical protein | -2.427 | 0.014881 |
| A5904_RS11690 | chalcone isomerase family protein | -1.721 | 0.015813 |
| novel0136 | hypothetical protein BAE28_07985 [Acidithiobacillus caldus] | -2.873 | 0.017295 |
| A5904_RS05745 | hypothetical protein | -1.951 | 0.017483 |
| novel0042 | hypothetical protein [Acidithiobacillus caldus] | -2.646 | 0.018786 |
| A5904_RS05845 | hypothetical protein | -1.972 | 0.019715 |
| novel0117 | hypothetical protein BAE30_00700, partial [Acidithiobacillus caldus] | -3.216 | 0.020525 |
| A5904_RS09755 | hypothetical protein | -2.251 | 0.022630 |
| novel0065 | 2-octaprenyl-6-methoxyphenyl hydroxylase [Acidithiobacillus caldus] | -3.177 | 0.023257 |
| A5904_RS01905 | hypothetical protein | -1.650 | 0.023562 |
| A5904_RS01380 | Lpp/OprI family alanine-zipper lipoprotein | -2.134 | 0.023755 |
| A5904_RS10710 | DUF4124 domain-containing protein | -1.776 | 0.024528 |
| A5904_RS04060 | hypothetical protein | -2.548 | 0.024599 |
| novel0187 | Slp family lipoprotein [Acidithiobacillus caldus] | -3.470 | 0.024853 |
| A5904_RS12380 | hypothetical protein | -2.373 | 0.027706 |
| novel0103 | prepilin-type N-terminal cleavage/methylation domain-containing protein [Acidithiobacillus caldus] | -2.756 | 0.029165 |
| A5904_RS05010 | Yip1 family protein | -2.022 | 0.032506 |
| A5904_RS15280 | hypothetical protein | -2.646 | 0.036223 |
| A5904_RS05695 | superinfection immunity protein | -2.282 | 0.036296 |
| A5904_RS15495 | SPOR domain-containing protein | -1.299 | 0.037940 |
| A5904_RS12550 | glycine zipper domain-containing protein | -2.111 | 0.040483 |
| A5904_RS07185 | hypothetical protein | -2.333 | 0.041434 |
| A5904_RS00490 | hypothetical protein | -1.801 | 0.042089 |
| novel0017 | hypothetical protein [Acidithiobacillus caldus] | -2.636 | 0.047771 |
| novel0150 | heavy metal-binding domain-containing protein [Acidithiobacillus caldus] | -2.462 | 0.048124 |
